# Co‐evolutionary adaptations of *Acinetobacter baumannii* and a clinical carbapenemase‐encoding plasmid during carbapenem exposure

**DOI:** 10.1111/eva.13441

**Published:** 2022-07-05

**Authors:** Linyue Zhang, Ying Fu, Linghong Zhang, Qingye Xu, Yunxing Yang, Jintao He, Sebastian Leptihn, Belinda Loh, Robert A. Moran, Willem van Schaik, Mark Alexander Toleman, Qiong Chen, Lilin Liu, Yunsong Yu, Xiaoting Hua

**Affiliations:** ^1^ Department of Infectious Diseases, School of Medicine Sir Run Run Shaw Hospital Zhejiang University Hangzhou China; ^2^ Key Laboratory of Microbial Technology and Bioinformatics of Zhejiang Province Hangzhou China; ^3^ Department of Clinical Laboratory, School of Medicine Sir Run Run Shaw Hospital Zhejiang University Hangzhou China; ^4^ Department of Clinical laboratory, School of Medicine Affiliated Hangzhou First People's Hospital Zhejiang University Hangzhou China; ^5^ School of Medicine University of Edinburgh Institute Zhejiang University Hangzhou China; ^6^ College of Medical and Dental Sciences Institute of Microbiology and Infection University of Birmingham Birmingham UK; ^7^ Department of Medical Microbiology, Division of Infection and Immunity Cardiff University Cardiff UK

**Keywords:** *Acinetobacter baumannii*, conjugative plasmid, experimental evolution, fitness, horizontal gene transfer, OXA‐23, plasmid‐host adaption

## Abstract

OXA‐23 is the predominant carbapenemase in carbapenem‐resistant *Acinetobacter baumannii*. The co‐evolutionary dynamics of *A. baumannii* and OXA‐23‐encoding plasmids are poorly understood. Here, we transformed *A. baumannii* ATCC 17978 with pAZJ221, a *bla*_OXA−23_‐containing plasmid from clinical *A. baumannii* isolate A221, and subjected the transformant to experimental evolution in the presence of a sub‐inhibitory concentration of imipenem for nearly 400 generations. We used population sequencing to track genetic changes at six time points and evaluated phenotypic changes. Increased fitness of evolving populations, temporary duplication of *bla*_OXA−23_ in pAZJ221, interfering allele dynamics, and chromosomal locus‐level parallelism were observed. To characterize genotype‐to‐phenotype associations, we focused on six mutations in parallel targets predicted to affect small RNAs and a cyclic dimeric (3′ → 5′) GMP‐metabolizing protein. Six isogenic mutants with or without pAZJ221 were engineered to test for the effects of these mutations on fitness costs and plasmid kinetics, and the evolved plasmid containing two copies of *bla*_OXA−23_ was transferred to ancestral ATCC 17978. Five of the six mutations contributed to improved fitness in the presence of pAZJ221 under imipenem pressure, and all but one of them impaired plasmid conjugation ability. The duplication of *bla*_OXA−23_ increased host fitness under carbapenem pressure but imposed a burden on the host in antibiotic‐free media relative to the ancestral pAZJ221. Overall, our study provides a framework for the co‐evolution of *A. baumannii* and a clinical *bla*_OXA−23_‐containing plasmid in the presence of imipenem, involving early *bla*_OXA−23_ duplication followed by chromosomal adaptations that improved the fitness of plasmid‐carrying cells.

## 1 INTRODUCTION

*Acinetobacter baumannii* is a Gram‐negative nosocomial pathogen that has acquired resistance to multiple antibiotics, making clinical therapy challenging (Wong et al., 2017). Carbapenem‐resistant *A. baumannii* (CRAB) mostly affects debilitated patients, resulting in increased morbidity, mortality, and healthcare costs (Hsu et al., 2017; Partridge et al., 2018). The emergence and prevalence of CRAB is a global public health threat because carbapenems are powerful, broad‐spectrum β‐lactams widely regarded as antibiotics of last resort. Overexpression of genes encoding oxacillinases (OXAs) is the most common carbapenem resistance mechanism in *A. baumannii* (Hamidian & Nigro, 2019; Wong et al., 2017). Enzymatic hydrolysis of carbapenems mediated by OXAs is slow. However, strong promotors provided by insertion sequence (IS) elements inserted immediately upstream of *bla*_OXA_ genes can lead to overexpression resulting in clinical resistance (Tietgen et al., 2019). Mobile *bla*_OXA_ genes bracketed by IS elements in composite transposons such as Tn*2006*, Tn*2007*, Tn*2008*, and Tn*2009* can be transferred horizontally and play an important role in the dissemination of carbapenem resistance in *A. baumannii* (Hamidian & Nigro, 2019; Yoon et al., 2017).

Plasmids harboring carbapenem resistance genes are important contributors to the spread of carbapenem resistance in *A. baumannii* (Chen et al., 2020; Kuo et al., 2018; Liu et al., 2021). The carbapenemase gene *bla*_OXA−23_ is predominant in Chinese CRAB isolates and the 77,530‐bp conjugative plasmid pAZJ221 is an important vector for horizontal transfer of *bla*_OXA−23_ (Liu et al., 2015). The 1164 bp replication initiation (*rep*) gene of pAZJ221 is identical to that of *A. baumannii* plasmid pABTJ1 (Huang et al., 2012), and pABTJ1 has been designated the reference plasmid for group GR25 under a typing scheme for *A. baumannii* plasmids based on the nucleotide identity of *rep* genes (Bertini et al., 2010; Salgado‐Camargo et al., 2020). pAZJ221 carries a complete set of genes associated with conjugation and the composite transposon Tn*2009*, which contains *bla*_OXA−23_ bracketed by IS*Aba1* (Liu et al., 2015). While many conjugative plasmids carry beneficial traits, they can exert physiological fitness costs‐of‐carriage resulting in reduced growth rates relative to plasmid‐free strains (Harrison & Brockhurst, 2012). A number of studies have demonstrated that these fitness costs can be compensated for through adaptive evolution, and compensation may be a key mechanism driving the spread of successful antibiotic‐resistant strains (Hall et al., 2021; MacLean & San Millan, 2019; Porse et al., 2016).

Extraordinarily high levels of antibiotic consumption in hospitals impose a strong selective pressure that drives the evolution and spread of antibiotic resistance determinants in nosocomial pathogens (San Millan, 2018). Thus, we hypothesized that plasmid–host adaptations between the clinically relevant plasmid pAZJ221 and its *A. baumannii* host would occur in the presence of a carbapenem. To elucidate the mechanisms of compensatory adaptations, the widely available and well‐characterized laboratory strain *A. baumannii* ATCC 17978 was used as a host (Ridenhour et al., 2017; Santos‐Lopez et al., 2019; Zlamal et al., 2021). We subjected *A. baumannii* ATCC 17978 containing pAZJ221 (ATCC 17978/pAZJ221 hereafter) to experimental evolution at a sub‐inhibitory concentration of imipenem for nearly 400 generations and applied population‐wide whole‐genome sequencing (WGS) to elucidate the dynamics of genomic adaptations. We constructed mutants to determine the role of specific mutations or an evolved variant of the plasmid in the co‐evolutionary process. Our work sheds light on the dynamics of the establishment of pAZJ221 in a new *A. baumannii* host and verified the effects of parallel chromosomal mutations and plasmid evolution on host fitness and plasmid characteristics.

## 2 MATERIALS AND METHODS

### 2.1 Bacterial strains and media

To construct the ancestral strain for the subsequent adaptive evolution experiment, we introduced the plasmid pAZJ221 to *A. baumannii* ATCC 17978 through electroporation. The transformed strain was called ATCC 17978/pAZJ221. Mueller Hinton (MH) Broth (Oxoid, UK) or Mueller Hinton (MH) Agar (Oxoid, UK) were used throughout this study.

### 2.2 Minimum inhibitory concentration (MIC) measurements

MIC assays were performed by the agar dilution method. Results of susceptibility testing were interpreted according to the Clinical and Laboratory Standards Institute guidelines (CLSI, 2021). *Escherichia coli* ATCC 25922 was included in all MIC assays as a control strain.

### 2.3 Preparation of electrocompetent cells

A fresh overnight culture was diluted 1:100 in 20 ml MH broth. Cells were grown at 37°C shaking at 200 rpm to logarithmic phase. Cells were harvested by centrifugation, suspended in 20 ml sterile H_2_O, and placed on ice for 30 min. Cells were collected by spinning for 3 min at 5000 rpm at 4 °C and washed with 20 ml 10% glycerol. This spinning and washing procedure was repeated three times. Cells were harvested by centrifugation and the supernatant was discarded, after which cells were suspended in 2 ml 10% glycerol, transferred to microfuge tubes (100 μl/tube), and stored at −70°C until use.

### 2.4 Electroporation

Electroporation was carried out using GenePulser XCell™ (Bio‐Rad, California, USA) and cuvettes with a 1 mm gap (Bio‐Rad). Electrocompetent cells stored at −70°C were thawed on ice. 200 ng plasmid DNA was added, and cells were transferred to the cuvette. Electroporation conditions were 1800 V, 25 μF, and 200 Ω. After electroporation, 1 ml MH broth was immediately added to the cuvette and the contents were transferred to sterile culture tubes for 1‐h growth, then the culture was spread on MH agar plates containing selective antibiotics, and the plates were cultured at 37 °C overnight. Transformants were verified by PCR and Sanger sequencing.

### 2.5 Evolutionary experiment

Three independent colonies of ATCC 17978/pAZJ221 were incubated in MH broth containing 16 mg/L imipenem and shaking (200 rpm) at 37°C for approximately 20 h. Every day, 20 μl overnight culture containing approximately 10^7^ cells was taken and passaged into 2 ml fresh media at a 1:100 dilution ratio. Three control groups were set for the mutation analysis, each containing four biological replicates: (i) ATCC 17978 without pAZJ221 under sub‐MIC imipenem pressure (0.0625 mg/L); (ii) ATCC 17978 without pAZJ221 in antibiotic‐free MH broth; (iii) ATCC 17978/pAZJ221 in antibiotic‐free MH broth. All populations were passaged at a 1:100 dilution ratio daily (i.e., approximately 6.6 generations per day) for a total of 60 days (approximately 400 generations in total) (Cooper, 2018). Every 10 days, stocks were generated for each population and stored frozen at −70°C.

### 2.6 Whole‐genome sequencing and sequence analyses

To identify the mutations in the populations and their allele frequencies, total DNA was extracted using a QIAamp DNA Minikit (Qiagen, Valencia, CA, USA) from a fresh culture inoculated from frozen stocks (see above). Eighteen populations of the experimental group and 71 populations of control groups were sequenced. Quality and quantity of genomic DNA were determined by visualization on agarose gels and spectroanalysis using a NanoDrop ND‐1000 (ThermoFisher, Waltham, MA, USA). A 300‐bp library for Illumina paired‐end sequencing was constructed using a Paired‐End DNA Sample Prep Kit (Illumina Inc.). The reads coverage of evolving populations on each reference sequence is listed in Table S1.

Mutations and their frequencies were determined using the software Breseq 0.27.1 with the default parameters and the ‐p flag (Deatherage & Barrick, 2014). IS element rearrangements in population samples were predicted by ijump (https://github.com/sleyn/ijump). Reference sequences were downloaded from NCBI (ATCC 17978‐mff: CP012004.1, pAB1: CP000522.1, pAB2: CP000523.1, pAB3: CP012005.1, pAZJ221: KM922672.1). We corrected the published ATCC 17978‐mff reference backbone for the variants that were detected in our ATCC 17978 strain (SRR12569044), which had been sequenced by Illumina paired‐end sequencing. Mutations were manually curated and filtered under the following criteria: (i) mutations were filtered if the gene or the intergenic region was found to contain an identical nucleotide mutation in any control group or the ancestor strain; (ii) mutations that did not reach a cumulative frequency of at least 20% across all populations were removed; (iii) mutations that did not change in frequency over time, indicating mis‐mapped reads or sequencing errors. The criteria were set in line with a published study (Scribner et al., 2020). Strains with parallel mutations were screened by isolating separate colonies from the corresponding evolving populations, and parallel mutations were verified by PCR and Sanger sequencing. Primers are listed in Table S2.

The locations of small RNAs (sRNAs) were predicted by “AcinetoCom” (Kröger et al., 2018), and RNA secondary structures were predicted by MXfold (Sato et al., 2021). Putative target mRNAs for sRNAs were predicted by TargetRNA2, and the COG categories of target genes were annotated by eggnog‐mapper (Cantalapiedra et al., 2021; Huerta‐Cepas et al., 2019; Kery et al., 2014). The conservation degree of a gene product among *A. baumannii* strains was determined using BLASTp to query the nonredundant protein sequences database on the NCBI server. Signal peptide and cleavage sites were predicted using SignalP 6.0 (Teufel et al., 2022). Conserved domains of proteins were analyzed by Conserved Domain Database (CDD) and SMART (Letunic et al., 2021; Marchler‐Bauer et al., 2017; Teufel et al., 2022). HHpred tool was employed for protein remote homology detection (Zimmermann et al., 2018). The interpretation of mutation effects and the molecular modeling were performed by AlphaFold 2.0 (Jumper et al., 2021).

To confirm the existence of a *bla*_OXA−23_ duplication, two single colonies were isolated from lineage 2 on day 10 and day 60, respectively. Strains were sequenced using the Nanopore MinION platform (Oxford Nanopore Technologies, Oxford, United Kingdom). Long‐read library preparation for Nanopore sequencing was performed with a 1D sequencing kit (SQK‐LSK109; Nanopore) without fragmentation. The libraries were then sequenced on a MinION device with a 1D flow cell (FIO‐MIN106; Nanopore) and base called with Guppy v2.3.5 (Nanopore). The long‐read data were used in a hybrid de novo assembly using Unicycler v0.4.8 (Wick et al., 2017).

### 2.7 Fitness evaluation

The growth curve, the area under the curve (AUC), and the maximum optical density (max OD) were used as indicators of fitness (San Millan, 2018). We defined a statistically significant advantage in at least one of the two parameters, AUC and max OD, as indicative of a fitness advantage. Three independent cultures per tested strain were grown overnight, diluted 1:200 in MH broth, and added to a flat‐bottom honeycomb 100‐well plate in three replicates. The plate was incubated at 37°C under agitation. OD_600_ of each culture was determined in 10‐min intervals for a total duration of 20 h using Bioscreen C Analyser (Oy Growth Curves Ab. Ltd.). Blank wells, which only contained the medium, were used to adjust for changes in OD_600_ not caused by microbial growth. Where required for the experiment, imipenem was added in MH broth to reach a final concentration of 16 mg/L. The max OD was regarded as the OD_600_ value determined at the time point of 1200 min. Growth curves were constructed and used to calculate AUC using GraphPad Prism 8.

### 2.8 Mutant construction

We modified the suicide vector pMo130‐Tel^R^, which contains a tellurite‐resistance marker and a *sacB* gene conferring sucrose sensitivity (Amin et al., 2013). We replaced the tellurite‐resistance marker with a hygromycin‐resistance marker and called the modified vector pMo130‐Hyg^R^. We used the modified vector to construct mutants as described previously (Hua et al., 2021). A 2‐kb fragment containing the mutation was generated by PCR and cloned into the *Bam*HI and *Xba*I site of pMo130‐Hyg^R^ using the ClonExpress® II One Step Cloning Kit (Vazyme Biotech Co., Ltd.) as previously described (Zhang et al., 2020). pMo130‐Hyg^R^ derivatives were introduced to *A. baumannii* ATCC 17978 by electroporation and would integrate into the chromosome by homologous recombination (first‐crossover). Transformants were selected on MH agar containing 100 mg/L hygromycin, where they turned yellow when sprayed with 0.45 M pyrocatechol. Transconjugants were cultured in LB broth containing 10% sucrose to select double recombinants, and serial dilutions were spread onto MH agar plates and sprayed with 0.45 M pyrocatechol to monitor excision of the suicide vector. The mutation was confirmed by PCR and Sanger sequencing using primers in Table S2.

### 2.9 Quantification of plasmid copy numbers and *bla*_OXA_ _−23_ copy numbers

The copy numbers of plasmids and *bla*_OXA−23_ were determined by quantitative polymerase chain reaction (qPCR) using a LightCycler^®^ 480 instrument (Roche Molecular Diagnostics). The experiment was performed by referring to San Millan et al. (2014). Three independent DNA extractions were performed per strain or population. DNA extractions were performed from 2 ml of MH broth cultures without antibiotics and using a QIAamp DNA Minikit (Qiagen). DNA was quantified using Qubit 3.0 (Invitrogen) following manufacturers' instructions. A previous study showed that accurate qPCR‐based copy number results would be obtained with total DNA extracts treated with a restriction enzyme that linearizes the plasmid (Providenti et al., 2006). Therefore, 2 μl DNA from each sample was digested using 2.5 μl FastDigest *Eco*RI (Thermo Fisher Scientific) for 30 min at 37°C. *Eco*RI was inactivated at 80°C for 5 min. No *Eco*RI targets are present in the amplification product of any reaction. We used pAZJ221 putative origin of replication gene *repA* and the *rpoD* chromosomal monocopy gene to compare the ratio of plasmid and chromosomal DNA.

To calculate primer efficiency, serial dilutions of ATCC 17978/pAZJ221 wild‐type *Eco*RI‐digested genomic DNA were used to create a standard curve for each pair of primers. The average cycle threshold (CT) was obtained for each point and plotted against the log_10_ of each concentration. The slope of the regression was obtained, and the efficiency of the primers was calculated using the following formula: (10^1/slope^ − 1) × 100% (Dorado‐Morales et al., 2021). Primer sequences, the efficiency of the reactions, the targets, and the size of amplicons are presented in Table S3.

qPCRs were performed using UltraSYBR Mixture (CWBIO, China) at a final DNA concentration of 0.25 ng/μl. The amplification conditions were as follows: initial denaturation for 10 min at 95°C, followed by 35 cycles of denaturation for 10 s at 95°C, annealing for 30 s at 55°C, and extension for 32 s at 72°C.

To calculate the plasmid copy number per chromosome, we used the formula:

$$\mathrm{cn}={\frac{{\left(1+E_{c}\right)}^{{\mathrm{Ct}}_{c}}}{{\left(1+E_{p}\right)}^{{\mathrm{Ct}}_{p}}}}^{}\times \;\frac{S_{c}}{S_{p}}$$

In this formula, cn is the plasmid copy number per chromosome, Sc and Sp are the sizes of the chromosomal and plasmid amplicons (in bp), Ec and Ep are the efficiencies of the chromosomal and plasmid qPCRs, and Ctc and Ctp are the threshold cycles of the chromosomal and plasmid reactions, respectively.

To calculate the copy number of *bla*_OXA−23_ per plasmid, we used the formula:

$$\mathrm{bn}=\frac{{\left(1+E_{p}\right)}^{{\mathrm{Ct}}_{p}}}{{\left(1+E_{b}\right)}^{{\mathrm{Ct}}_{b}}}\times \frac{S_{p}}{S_{b}}$$

In this formula, bn is the *bla*_OXA−23_ copy number per plasmid, Sb is the sizes of *bla*_OXA−23_ amplicons (in bp), Eb is the efficiency of *bla*_OXA−23_ qPCRs, and Ctb is the threshold cycles of *bla*_OXA−23_ reactions.

### 2.10 Plasmid stability determination

We assessed the stability of pAZJ221, in the absence of selective pressure, in the constructed mutants and in ATCC 17978 after 100 generations of propagation. Three biological replicates of each strain were cultured in MH broth. Every day, the culture of each strain was diluted 1000‐fold into 2 ml of fresh MH broth without antibiotics (about 10 generations per day). Cultures were incubated at 37°C with 200 rpm shaking. On day 10 (~100 generations), each culture was diluted and plated on MH agar without antibiotics. Fifty colonies of each culture were replicated on MH agar containing 8 mg/L meropenem. ATCC 17978 and ATCC 17978/pAZJ221 were used as negative and positive controls, respectively, to ensure the effectiveness of meropenem. The proportion of bacteria harboring pAZJ221 was determined by the number of colonies growing on meropenem‐containing MH agar divided by 50.

### 2.11 Conjugation rate and conjugation frequency

We measured the conjugation rates and conjugation frequencies (the number of transconjugants per recipient) to assess plasmid conjugation ability. The conjugation rate is expressed as mL cell^−1^ h^−1^ (Alderliesten et al., 2020). Conjugation between plasmid‐containing donors and plasmid‐free recipients was measured in antibiotic‐free MH broth as previously described by Simonsen et al. (1990). In this study, the donor is wild‐type ATCC 17978/pAZJ221 or mutant/pAZJ221 or wild‐type ATCC 17978/pAZJ221E, and the recipient is a rifampin‐resistant ATCC 17978. The donor and recipient cultures were grown overnight. 5 μl of each overnight well‐mixed culture was inoculated together into a tube containing 5 ml MH broth and incubated at 37°C with 200 rpm for 24 h. OD_600_ of the combined culture after incubating for 3 and 4 hours was measured to calculate the maximum growth rate for the combined culture during the exponential phase. The overnight mixed culture was diluted and spread onto appropriate agar media. We calculated the number of colonies (CFU/mL) that could grow on antibiotic‐free MH agar (N), 8 mg/L meropenem MH agar (A), 50 mg/L rifampin MH agar (B), and 8 mg/L meropenem plus 50 mg/L rifampin MH agar (T). The number of donors (D) = A‐T, and the number of recipients (R) = B. The cell densities were then used to calculate the conjugation rate λ (in ml cell^−1^ h^−1^) as follows:

$$\lambda =\varphi \;\ln\left(1+\frac{T}{R}\cdot \frac{N}{D}\right)\frac{1}{N}$$

ᵠ is the maximum growth rate of mixed donor and recipient cultures, which is calculated from OD_600_ measured at two time point (hour), *t*_*a*_ and *t*_*b*_, during the exponential phase:

$$\varphi =\frac{\ln\left({\mathrm{OD}}_{b}/{\mathrm{OD}}_{a}\right)}{t_{b}-t_{a}}$$

Another parameter conjugation frequency (T/R) was also evaluated using the same stationary phase cell culture.

### 2.12 Statistical analysis

Statistical analyses were performed following the principles below. For comparison between two samples, we used the D'Agostino & Pearson test for normal distribution and the F test for equality of variances. If the data passed the normality test (alpha = 0.01), we performed unpaired *t*‐tests with Welch's correction for unequal variances. If the data did not follow a normal distribution, we performed the Mann–Whitney U test. For multiple sample comparisons, we used the D'Agostino & Pearson test for normal distribution and the Brown–Forsythe test for equality of variances. If the data passed the normality test (α= 0.01), we performed one‐way ANOVA test and Dunnett's multiple comparisons test with Dunnett's T3 multiple comparisons test for unequal variances. If the data did not follow a normal distribution, we performed Kruskal–Wallis test and Dunn's multiple comparisons test. All analysis was performed by GraphPad Prism 8.

## 3 RESULTS

### 3.1 Evolving populations displayed fitness advantages relative to the ancestor

Across three biological replicates, 97.33 ± 1.15% of examined ATCC 17978 cells in MH broth retained pAZJ221 after approximately 100 generations, indicating that the plasmid was stably maintained by ATCC 17978 in the absence of selective pressure. The conjugation frequency of pAZJ221 from ATCC 17978 to a rifampin‐resistant ATCC 17978 was determined to be 4.08 × 10^−1^ ± 1.44 × 10^−1^ transconjugants per recipient in overnight liquid cultures. It should be noted that other studied *Acinetobacter* plasmids did not exhibit conjugation frequencies this high when frequencies were calculated as transconjugant per recipient (Mindlin et al., 2021), which cannot be compared with frequencies calculated as transconjugant per donor (H. Liu et al., 2021; Zong et al., 2020) (Table S4).

**FIGURE 1:**
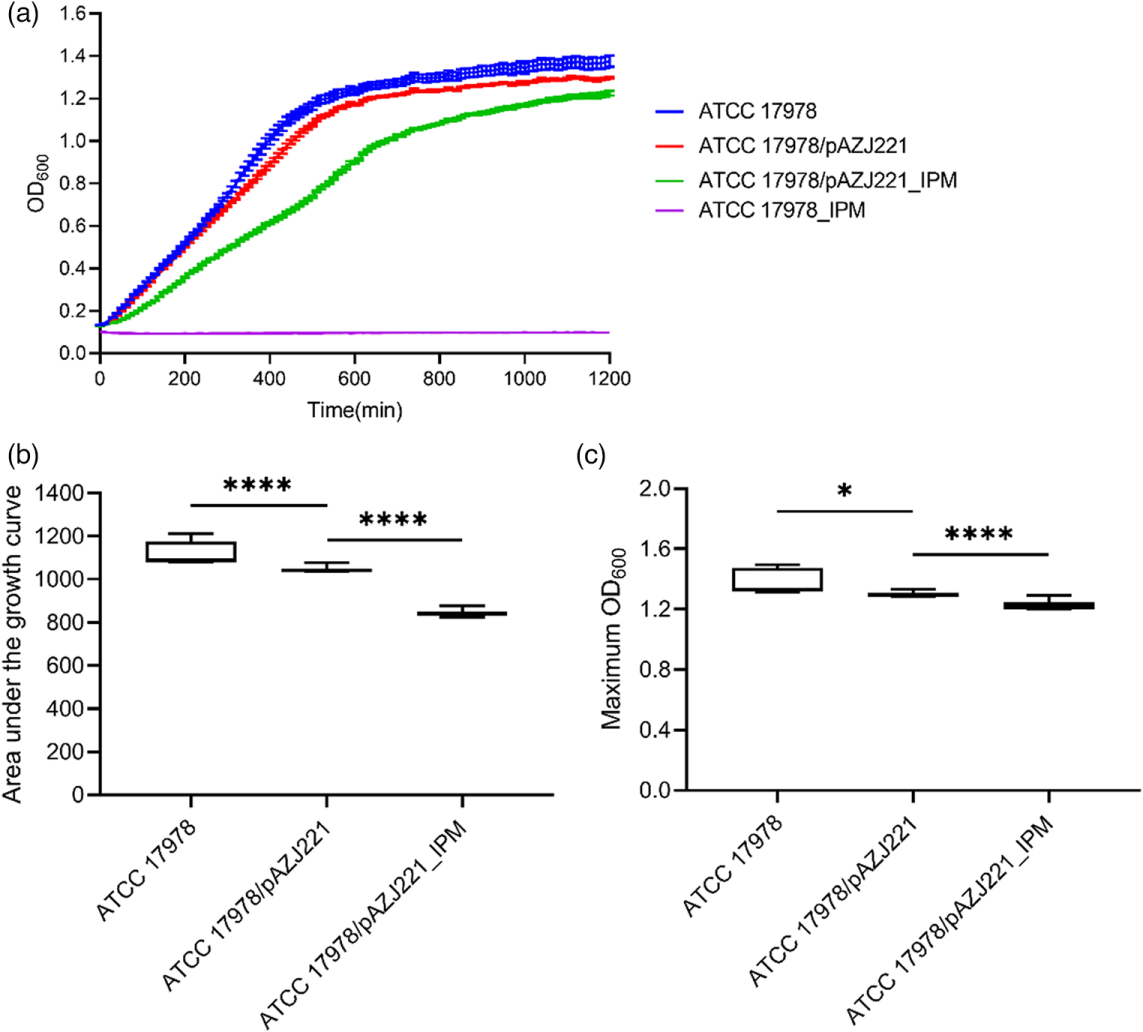
Fitness cost of pAZJ221 in *A. baumannii* ATCC 17978 with or without imipenem pressure. The growth curve (a), the area under the curve (b), and the maximum OD_600_ (c) of ATCC 17978 and ATCC 17978/pAZJ221 in MH broth and imipenem (IPM)‐containing MH broth were assessed and compared. ATCC 17978 could not grow in IPM‐containing MH broth, pAZJ221 imposed a fitness cost on ATCC 17978, and ATCC 17978/pAZJ221 exhibited significantly decreased fitness under sub‐MIC imipenem pressure compared with the antibiotic‐free environment. Box plots show median, lower, and upper quartiles, the minimum value, and the maximum value. Data were collected for 20 h at 37°C with shaking. Statistical analyses were performed through unpaired *t*‐tests with Welch's correction for unequal variances. When the data did not follow normal distribution, Mann–Whitney test was performed. *p < 0.05; ****p < 0.0001

The apparent high conjugation rate of pAZJ221 complicates estimates of the physiological burden of plasmid carriage in direct competition assays, because costs can be masked by the consequences of plasmid transmission into the competing strain. Therefore, we measured the effect of pAZJ221 on the fitness of ATCC 17978 by assessing three parameters of bacterial growth: the growth curve, AUC, and max OD. We confirmed that pAZJ221 imposed a fitness cost on ATCC 17978, and ATCC 17978 containing pAZJ221 exhibited significantly decreased fitness in the presence of sub‐MIC imipenem relative to antibiotic‐free conditions (Figure 1). Thus, we designed an evolutionary experiment to investigate compensatory host‐plasmid co‐evolution that alleviates the cost of pAZJ221 in the presence of a carbapenem. The experimental setup is shown in Table 1.

**TABLE 1:** Experimental setup of the experimental group and control groups

Variable	Experimental group	Control group 1	Control group 2	Control group 3
Media	MH broth	MH broth	MH broth	MH broth
IPM concentration (mg/L)	16	0	0.0625	0
ATCC 17978	+^a^	+	+	+
pAZJ221	+	+	−^b^	−
Number of biological replicates	3	4	4	4

Abbreviation: IPM, imipenem.

^a^ “+” means that the corresponding variable exists.

^b^ “−” means that the corresponding variable does not exist.

**FIGURE 2:**
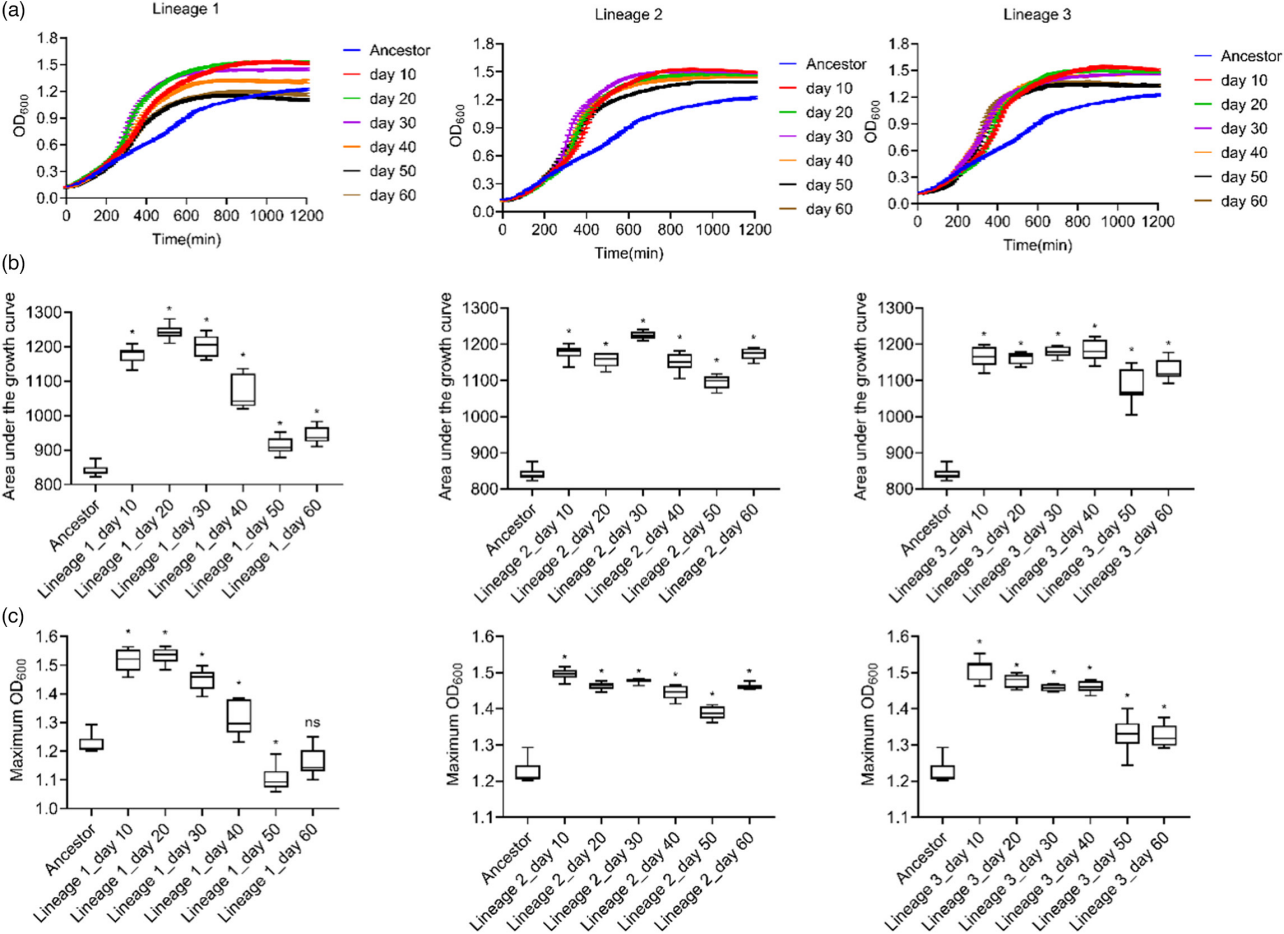
Fitness of evolving populations at 6 time points from lineage 1, 2, and 3. The growth curve (a), the area under the curve (b), and the maximum OD_600_ (c) of each population were compared with the same data of ancestral strain ATCC 17978/pAZJ221 in imipenem‐containing MH broth. Data were collected for 20 h at 37 °C with shaking. Statistical analysis was performed through Dunnett's multiple comparisons test with Dunnett's T3 multiple comparisons test for unequal variances. *, adjusted *p* value < 0.05; ns, not statistically significant

Three experimental lineages were propagated. For each, a population was stored from each of six time points (day 10, 20, 30, 40, 50, and 60) such that each lineage was represented by six sampled populations. We measured growth curves, AUCs, and max ODs in the evolution environment as fitness proxies both for the ancestor ATCC 17978/pAZJ221 and 18 (three lineages × six time points) independently evolved populations. The triplicate parallel lineages were assigned numbers from 1 to 3. The evolved populations were named after their lineage (1, 2, or 3) followed by the storage time point (day 10, 20, 30, 40, 50, or 60). Significant advantages on growth curves and statistically higher AUCs were observed in all populations compared with the ancestral strain ATCC 17978/pAZJ221 (all adjusted *p* value <0.05) (Figure 2). In lineage 1, the max OD on day 50 was lower than that of the ancestral strain, possibly due to biofilm formation in the later stages of the experiment (Figure S1).

### 3.2 Dynamic allele frequency changes

To determine the genetic bases for the observed evolutionary adaptations, we conducted whole‐population WGS of evolved populations, control populations, and the ancestral clone to track allele frequency dynamics. We found that adaptations might be the result of mutations in the form of single‐nucleotide polymorphisms (SNPs) within genes or in intergenic regions. We defined a “genuine” mutation as one that only occurred in the experimental group. If the same SNP was observed in both the experimental group and the control groups, we did not regard it as a mutation that arose in response to the presence of pAZJ221 and imipenem. In total, we detected 26 genuine chromosomal point mutations: 65.4% (17/26) were nonsynonymous, and 34.6% (9/26) were in intergenic regions. Of nine SNPs in intergenic regions, five were predicted to occur in sRNAs.

We observed dynamic changes of allele frequencies in all lineages. The frequencies of some mutations initially increased before decreasing and disappearing, while other mutations appeared and gradually became dominant. Different mutations competed in the same lineage, supporting the idea of clonal interference (Gerrish & Lenski, 1998; Hughes et al., 2012; Lang et al., 2011). A complete list of mutations detected in the experimental group and control groups is provided in Table S5, and the allele frequencies of genuine mutations in the experimental group are shown in Figure S2.

### 3.3 Mutations on parallel targets

Parallelism at the level of nucleotides, amino acids, genes, or pathways in replicate populations is strong evidence of selection on that target and might suggest that these alterations provide an ideal adaptive solution (Cooper, 2018). In our study, parallel evolution at the single‐nucleotide level was not observed among lineages of the experimental group. Thus, we focused on mutations that appeared in parallel loci across all three evolving lineages.

Locus‐level parallelism was identified in four loci, comprising regions upstream of two open reading frames (ORFs) (ACX60_07155 and AC60_11780) and within two ORFs (ACX60_08060 and ACX10935). ACX60_11780 and ACX60_07155 encode putative TetR family transcriptional regulators and will hereafter be referred to as *tetR1* and *tetR2*, respectively. The four SNPs located upstream of *tetR1* and *tetR2* were predicted to occur in sRNAs. ACX60_08060 encodes a cyclic dimeric (3′ → 5′) GMP (c‐di‐GMP) ‐metabolizing GGDEF and EAL domain‐containing protein and will hereafter be called *abDGC1*. ACX60_10935 was predicted to encode a DcaP‐like membrane protein in *A. baumannii* by the HHpred tool (Gabler et al., 2020) and will hereafter be called *dcaP2*.

### 3.4 Mutations in sRNAs

The details and acronyms of mutations in parallel loci are provided in Table 2 to facilitate the presentation of the results. Two different SNPs, C‐424 T and G‐339 T, arose upstream of *tetR1* in lineage 2 and lineage 3, respectively. Two sRNAs were identified upstream of *tetR1*, sRNA66 on the negative strand and sRNA67 on the positive strand. MXfold predictions suggested that the *tetR1*(C‐424 T) mutation would shorten a stem in sRNA67 and *tetR1*(G‐339 T) would change the secondary structure of sRNA66 (Figure S3). Mutations C‐378A and G‐399A upstream of *tetR2* were found in lineage 1 and lineage 3. A single sRNA, sRNA33, was found on the positive strand upstream of *tetR2*. The *tetR2*(C‐378A) mutation was predicted to change the secondary structure of sRNA33, while *tetR2*(G‐399A) would increase the size of the hairpin loop in sRNA33 (Figure S4).

**TABLE 2:** List of mutations that occur at the same gene locus or intergenic region among lineages of the experimental group

Source	Gene locus	Mutation	Annotation	Genotype acronym
Lineage 2	ACX60_11780/ACX60_11785	intergenic (−424/+580) C → T	TetR/AcrR family transcriptional regulator / DnaJ domain‐containing protein	*tetR1*(C‐424 T)
Lineage 3	ACX60_11780/ACX60_11785	intergenic (−339/+665) G → T	TetR/AcrR family transcriptional regulator / DnaJ domain‐containing protein	*tetR1*(G‐339 T)
Lineage 1	ACX60_08060	D589Y (GAT→TAT)	c‐di‐GMP‐metabolizing GGDEF and EAL domains	*abDGC1*(D589Y)
Lineage 2	ACX60_08060	D341N (GAT→AAT)	c‐di‐GMP‐metabolizing GGDEF and EAL domains	*abDGC1*(D341N)
Lineage 1	ACX60_07150/ACX60_07155	intergenic (+561/−378) C → A	hypothetical protein / TetR/AcrR family transcriptional regulator	*tetR2*(C‐378A)
Lineage 3	ACX60_07150/ACX60_07155	intergenic (+540/−399) G → A	hypothetical protein / TetR/AcrR family transcriptional regulator	*tetR2*(G‐399A)
Lineage 1	ACX60_10935	Q6* (CAA → TAA)	Hypothetical protein (probably DcaP‐like membrane protein)	*dcaP2*(Q6*)
Lineage 1	ACX60_10935	W77* (TGG → TAG)	Hypothetical protein (probably DcaP‐like membrane protein)	*dcaP2*(W77*)
Lineage 2	ACX60_10935	D94N (GAT→AAT)	Hypothetical protein (probably DcaP‐like membrane protein)	*dcaP2*(D94N)
Lineage 3	ACX60_10935	R174* (AGA → TGA	Hypothetical protein (probably DcaP‐like membrane protein)	*dcaP2*(R174*)

Putative target mRNAs for sRNA33, sRNA66, and sRNA67 were predicted. The results are listed in Table S6 and Figure S5. sRNA33 could affect genes involved in transcription (5/30), unknown functions (4/30), cell wall/membrane/envelope biogenesis (4/30), and energy production and conversion (3/30). sRNA66 could affect mRNAs predicted to be involved in energy production and conversion (3/25), amino acid transport and metabolism (3/25), cell wall/membrane/envelope biogenesis (2/25), inorganic ion transport and metabolism (2/25), and translation, ribosomal structure and biogenesis (2/25). sRNA67 could affect genes with unknown functions (11/50), amino acid transport and metabolism (7/50), transcription (4/50), cell wall/membrane/envelope biogenesis (4/50), and energy production and conversion (4/50).

### 3.5 Mutations in open reading frames

Two nonsynonymous mutations in *abDGC1*, D589Y and D341N, occurred in lineage 1 and lineage 2. C‐di‐GMP is one of the most common and important secondary messengers in bacteria metabolic systems (Ahmad et al., 2020). A BLASTp search with AbDGC1 amino acid sequence found homologues (>90% identity with ≥90% query coverage) in 266 *A. baumannii* strains. AbDGC1 consisted of a Per‐ARNT‐Sim (PAS) domain, a GGDEF domain, and an EAL domain. Essentially, c‐di‐GMP is synthesized by the diguanylate cyclase (DGC) activity of the GGDEF domain and degraded into two GMP molecules by the phosphodiesterase (PDE) activity of the EAL domain (Romling et al., 2013). The point mutation D341N (GAT→AAT) is in the GGDEF motif while another point mutation D589Y is in the EAL domain.

Based on the BLASTp results, 325 homologues (>90% identity with ≥90% query coverage) of DcaP2 were found in *A. baumannii* genomes and they were annotated to be hypothetical proteins. A signal peptide (Sec/SPI) was detected with a likelihood of 0.9025 by SignalP 6.0, and the cleavage site was between position 26 and 27; the signal peptide was removed from the amino acid sequence before any further analysis. No conserved domain was identified using NCBI's conserved domain database and SMART. HHPred tool predicted DcaP2 was a remote homologue of DcaP porin in *A. baumannii* with the estimated probability being 99.89%. Three nonsense mutations and one missense mutation D94N were detected within *dcaP2*. The nonsense mutations (Q6*, W77*, and R174*) were expected to render DcaP2 nonfunctional. We modeled the structures of wild‐type DcaP2 and DcaP2(D94N), and no significant impact on the structure was observed but the effect of the mutation on protein function requires further investigation (Figure S6). Thus, for the remainder of this study, we focused on mutations in sRNAs and in *abDGC1*.

### 3.6 Carbapenem MICs of the ancestor and mutants were indistinguishable

To address whether mutations on sRNAs and within *abDGC1* played a role in the adaptation of ATCC 17978 to pAZJ221, we introduced six mutations (listed in Table 3) to the original ATCC 17978 and constructed a range of mutant/plasmid combinations. No statistically significant differences in imipenem or meropenem MICs were observed in mutants relative to ATCC 17978/pAZJ221 (within a maximum of onefold difference) (Table S7). This indicates that the mutations do not contribute to carbapenem resistance levels. As the ancestral ATCC 17978/pAZJ221 was already carbapenem‐resistant, improved fitness may have been more important than the extent of resistance in these experiments. Thus, we evaluated the fitness of constructed mutants.

**TABLE 3:** Effects of six mutations from the experimental group

Genotype acronym	Predicted impact on the sRNA secondary structure or the gene function	Confer fitness advantage for ATCC 17978	Confer fitness advantage in the presence of pAZJ221 without antibiotic	Confer fitness advantage in the presence of pAZJ221 under imipenem pressure	Conjugation ability of pAZJ221 in mutants	Stability of pAZJ221 in mutants
*tetR1*(C‐424 T)	Shorten stem in sRNA67	Yes	Yes	Yes	Decreased	Decreased
*tetR1*(G‐339 T)	Changed secondary structure of sRNA66	Yes	Yes	Yes	Decreased	Decreased
*abDGC1*(D589Y)	Impaired PDE activity	No	Yes	Yes	NS	NS
*abDGC1*(D341N)	Impaired DGC activity	No	No	Yes	Decreased	NS
*tetR2*(C‐378A)	Changed secondary structure of sRNA33	No	No	No	Decreased	NS
*tetR2*(G‐399A)	Larger hairpin loop in sRNA33	No	No	Yes	Decreased	NS

Abbreviations: DGC, diguanylate cyclase; NS, not statistically significant; PDE, phosphodiesterase.

### 3.7 Diverse fitness effects of chromosomal parallel mutations

Our study involved two variables: the presence of pAZJ221 and the presence of imipenem pressure. Thus, we obtained the growth curves of strains under three conditions to elucidate whether, and under which conditions, these six mutations could confer advantages to the host.

**FIGURE 3:**
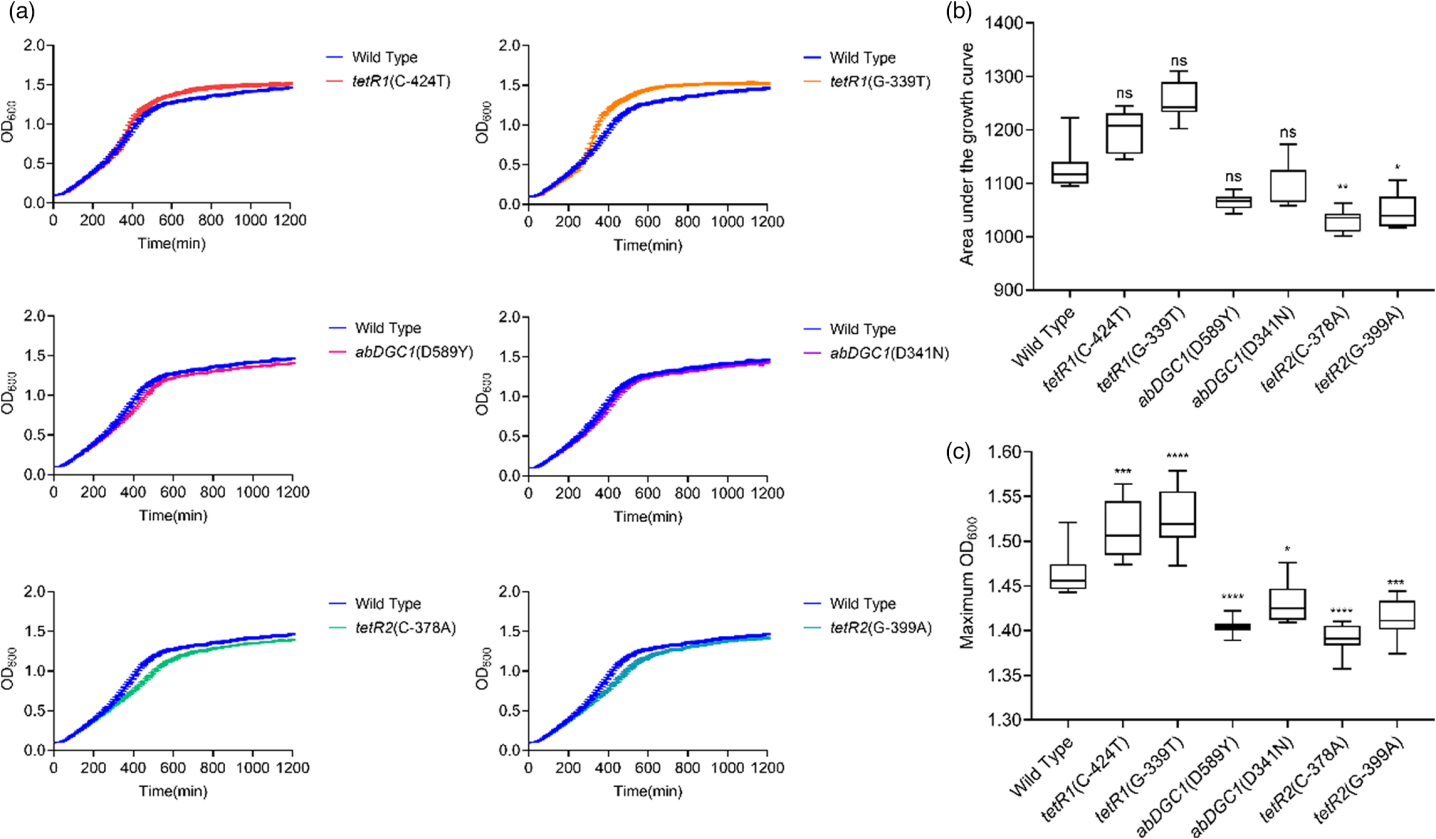
Growth curve (a), the area under the curve (b), and the maximum OD_600_ (c) of ATCC 17978 and 6 constructed mutants without pAZJ221 in antibiotic‐free MH broth. Box plots show median, lower, and upper quartiles, the minimum value, and the maximum value. Data were collected for 20 h at 37°C with shaking. Statistical analysis was performed through Dunnett's multiple comparisons test. When the data do not follow normal distribution, Dunn's multiple comparisons test was performed. *, adjusted *p* value < 0.05; **, adjusted *p* value < 0.01; ***, adjusted *p* value < 0.001;****, adjusted *p* value < 0.0001; ns, not statistically significant. Wild Type, ATCC 17978

For the first condition, we compared the growth curves, AUCs, and max ODs of the wild‐type ATCC 17978 and six constructed isogenic mutants harboring mutations listed in Table 3. These strains do not carry pAZJ221 and were tested in antibiotic‐free MH broth as they are carbapenem‐susceptible. The results showed that two mutations upstream of *tetR1* were beneficial, exhibiting significantly higher max OD values than the wild‐type strain (Figure 3).

**FIGURE 4:**
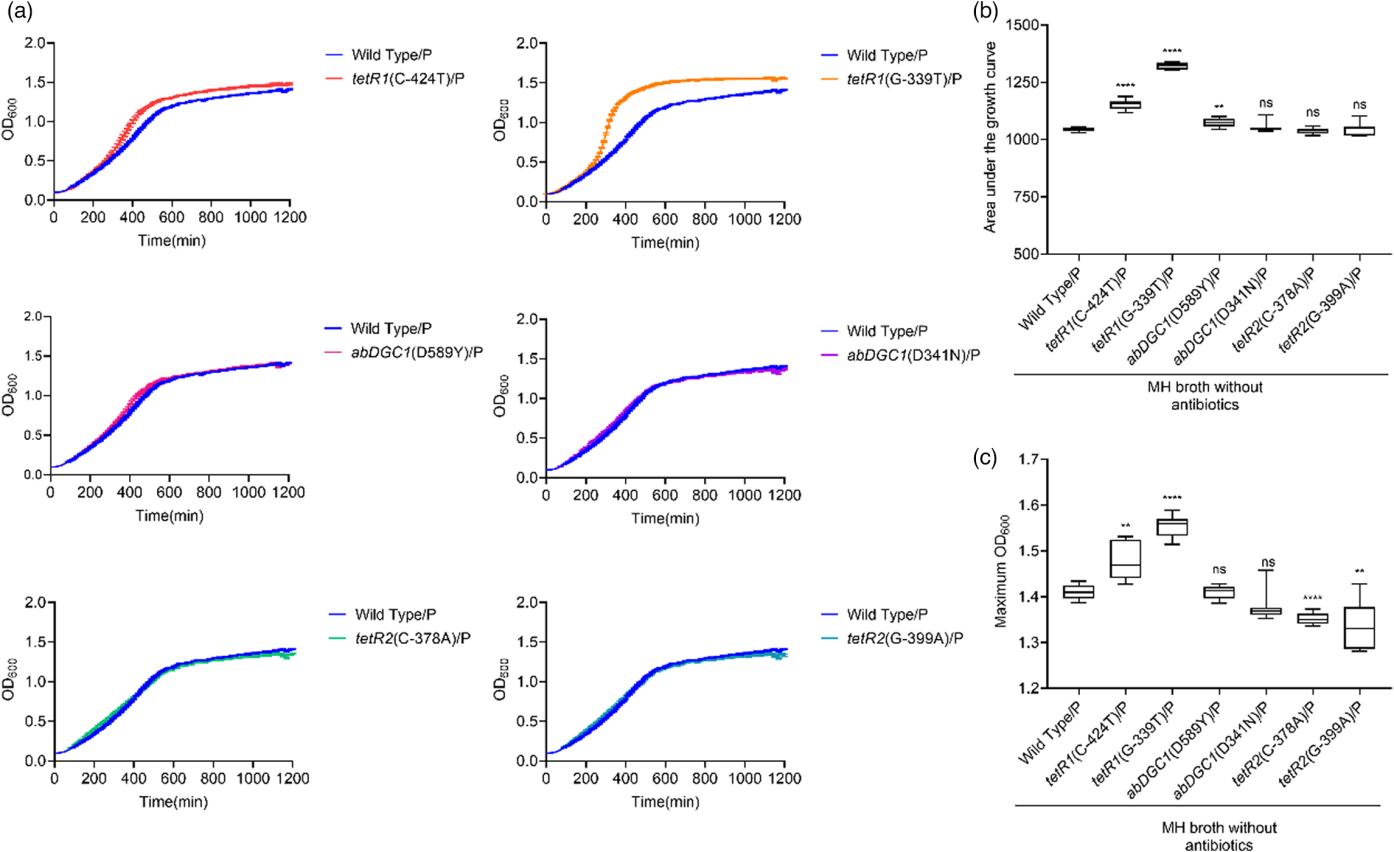
Fitness effects of mutations in the presence of pAZJ221 in antibiotic‐free MH broth. The growth curve (a), the area under the curve (b), and the maximum OD_600_ (c) were analyzed as fitness indicators. Box plots show median, lower, and upper quartiles, the minimum value, and the maximum value. Data were collected for 20 h at 37°C with shaking. Statistical analysis was performed through Dunnett's multiple comparisons test with Dunnett's T3 multiple comparisons test for unequal variances. When the data do not follow normal distribution, Dunn's multiple comparisons test was performed. **, adjusted *p* value < 0.01; ****, adjusted *p* value <0.0001; ns, not statistically significant; P, pAZJ221; Wild Type/P, ATCC 17978/pAZJ221

**FIGURE 5:**
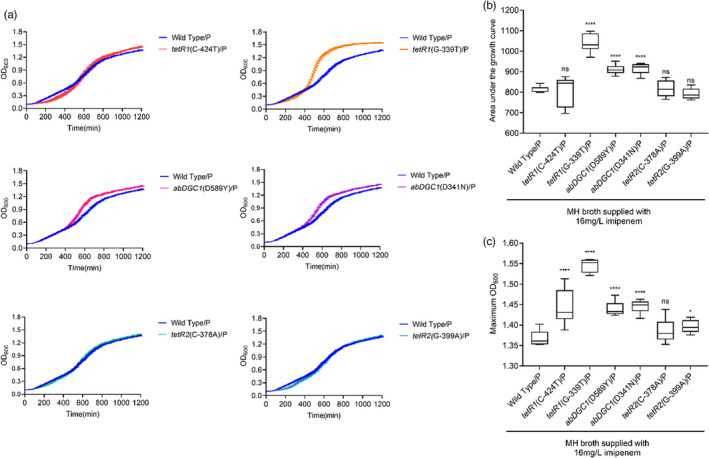
Growth curve (a), the area under the curve (b), and the maximum OD_600_ (c) of the ancestor strain ATCC 17978/pAZJ221 and 6 mutants with pAZJ221 were assayed in their evolved environment where 16 mg/L imipenem was supplied in MH broth. Box plots show median, lower, and upper quartiles, the minimum value, and the maximum value. Data were collected for 20 h at 37°C with shaking. Statistical analysis was performed through Dunnett's multiple comparisons test. *, adjusted *p* value < 0.05; ****, adjusted *p* value < 0.0001; ns, not statistically significant. P, pAZJ221; Wild Type/P, ATCC 17978/pAZJ221

We transformed pAZJ221 into the wild‐type ATCC 17978 and six constructed mutants and then obtained growth curves in antibiotic‐free MH broth (Figure 4) and 16 mg/L imipenem MH broth (Figure 5), respectively. Mutations upstream of *tetR1* still conferred fitness advantages in the presence of pAZJ221 with or without imipenem pressure. The mutation *tetR2*(C‐378A) did not provide any fitness advantage under all three conditions at the resolution of the growth curve experiment, which means that it is a neutral mutant allele under the tested conditions. Mutations in *abDGC1* had diverse effects on fitness, *abDGC1*(D589Y)‐producing ATCC 17978 exhibited a slightly higher AUC value than wild‐type in the presence of pAZJ221 in MH broth, but its max OD value was not significantly different from its wild‐type counterpart. When growing in the presence of imipenem, the AUC and max OD values of *abDGC1*(D589Y)/pAZJ221 were all higher than those of wild‐type/pAZJ221, indicating a positive fitness effect under imipenem pressure. As for the mutation *abDGC1*(D341N) and *tetR2*(G‐399A), higher fitness was only observed when pAZJ221‐containing mutants were subject to imipenem pressure.

The effects of mutations are summarized in Table 3. The pattern of fitness contribution indicated three strategies to mitigate the fitness cost imposed by pAZJ221 and imipenem exposure: (i) *tetR1*(C‐424 T) and *tetR1*(G‐339 T): “piggybacking” on the host adaptive mutations, the cost imposed by plasmid or carbapenem was relieved as a collateral cost‐mitigating effect of host adaptation; this strategy was also reported by a recent study (Kloos et al., 2021). Other mutations located upstream of *tetR1* were also detected in the control groups, including SNPs and 1‐bp deletion (Table S5); (ii) *abDGC1*(D589Y): plasmid‐specific compensatory mutations, this mutation could confer fitness advantages for the host in the presence of pAZJ221; (iii) *abDGC1*(D341N) and *tetR2*(G‐399A): evolution condition‐dependent compensatory mutations, higher fitness was only achieved when both pAZJ221 and imipenem pressure were present. Overall, five out of six mutations improved fitness in the presence of pAZJ221 under imipenem pressure.

### 3.8 Plasmid and *bla*_OXA_ _−23_ copy number changes during experimental evolution

**FIGURE 6:**
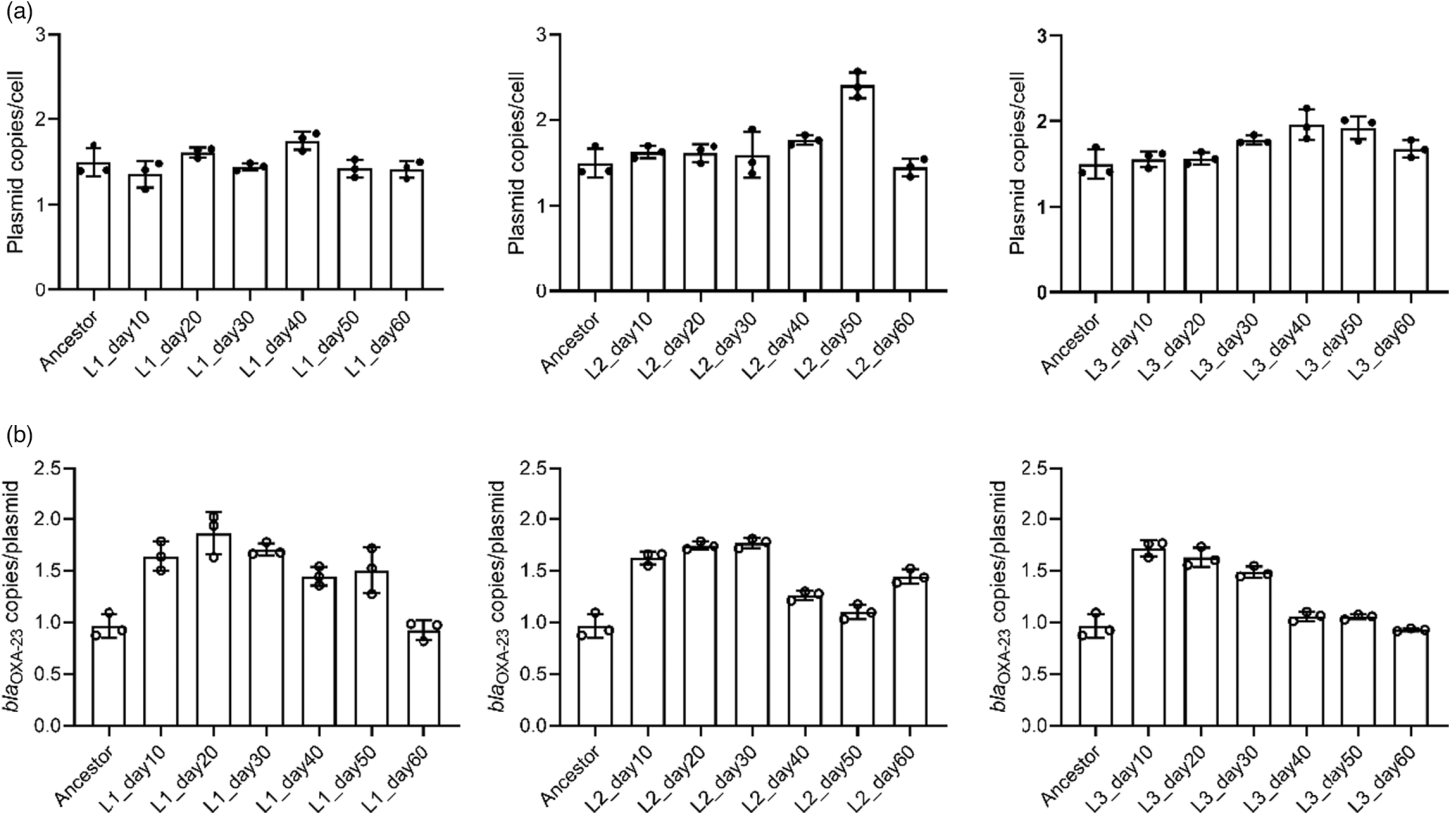
Changes of plasmid copy number per cell (a) and *bla*_OXA−23_ copy number per plasmid (b) in three lineages during evolution. The data are presented as mean values of the copy numbers from three independent experiments for each population at the corresponding time point. Error bars depict the standard deviation of the mean, and the dots represent the data of each experiment. Ancestor, ATCC 17978/pAZJ221; L, lineage

Since no SNPs were detected in pAZJ221 over the course of experimental evolution experiments, we assessed the population‐level dynamics of its basic characteristics, including copy number per cell and *bla*_OXA−23_ copy number per plasmid, in each evolving population. The copy number of pAZJ221 in ATCC 17978, determined by quantitative polymerase chain reaction (qPCR), is 1.50 ± 0.17. In evolving populations, the copy number of pAZJ221 ranged from 1.36 ± 0.16 (lineage1_day10) to 2.40 ± 0.15 (lineage2_day50) (Figure 6a).

The copy number of *bla*_OXA−23_ was 0.963 ± 0.116 per plasmid. Relative to the parental strain, duplication of *bla*_OXA−23_ was observed in all lineages in the early stages of evolutionary experiments, before its copy number decreased to the original level in lineages 1 and 3. The copy number of *bla*_OXA−23_ rose to 1.44 ± 0.07 in lineage 2 at the end of the experiment, following a slight decline (Figure 6b).

**FIGURE 7:**
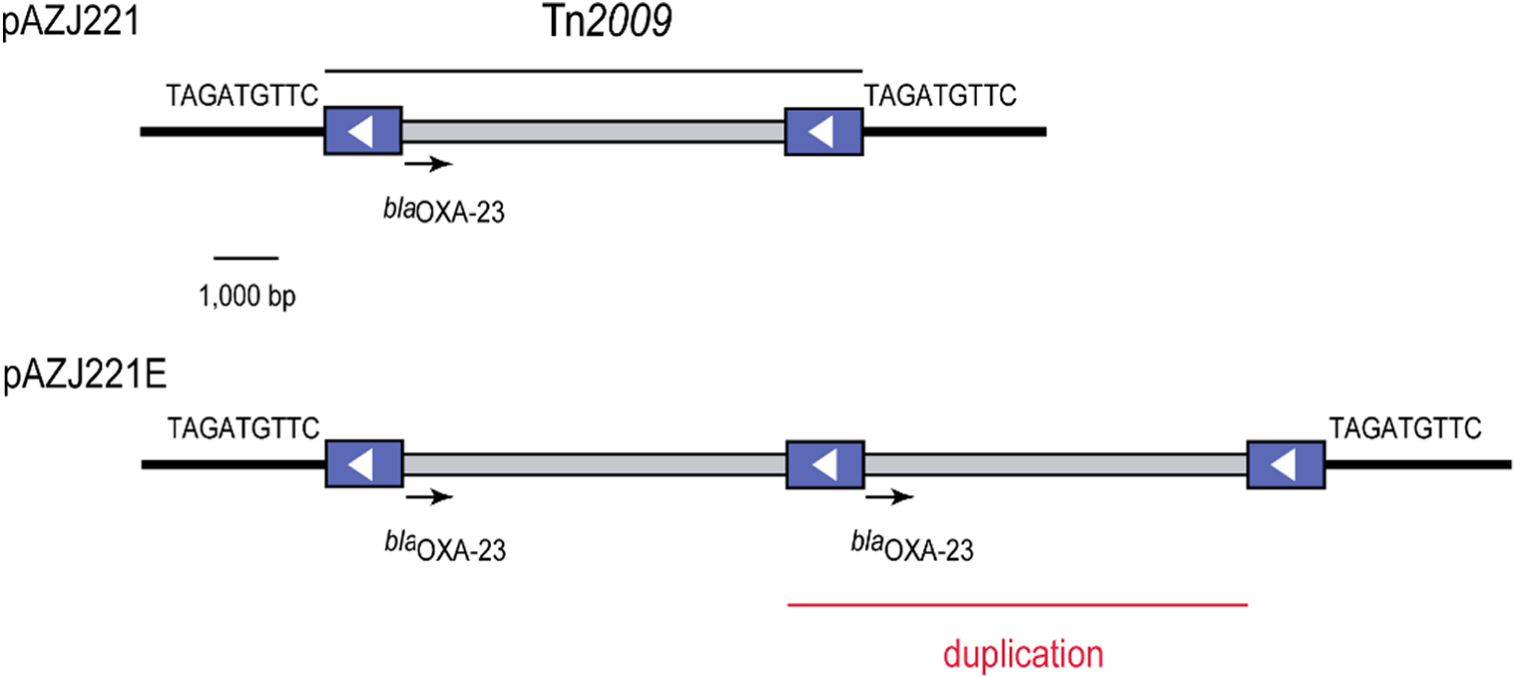
Structure of Tn*2009* in pAZJ221 and the Tn*2009* region featuring a partial duplication in pAZJ221E. The blue boxes represent IS*Aba1* with the white arrow indicating the orientation of its transposase. The sequences of direct repeats flanking Tn*2009* are shown. The black arrow indicates the extent and orientation of *bla*_OXA−23_. The sequence that has been duplicated in pAZJ221E is indicated by a labeled red line

**FIGURE 8:**
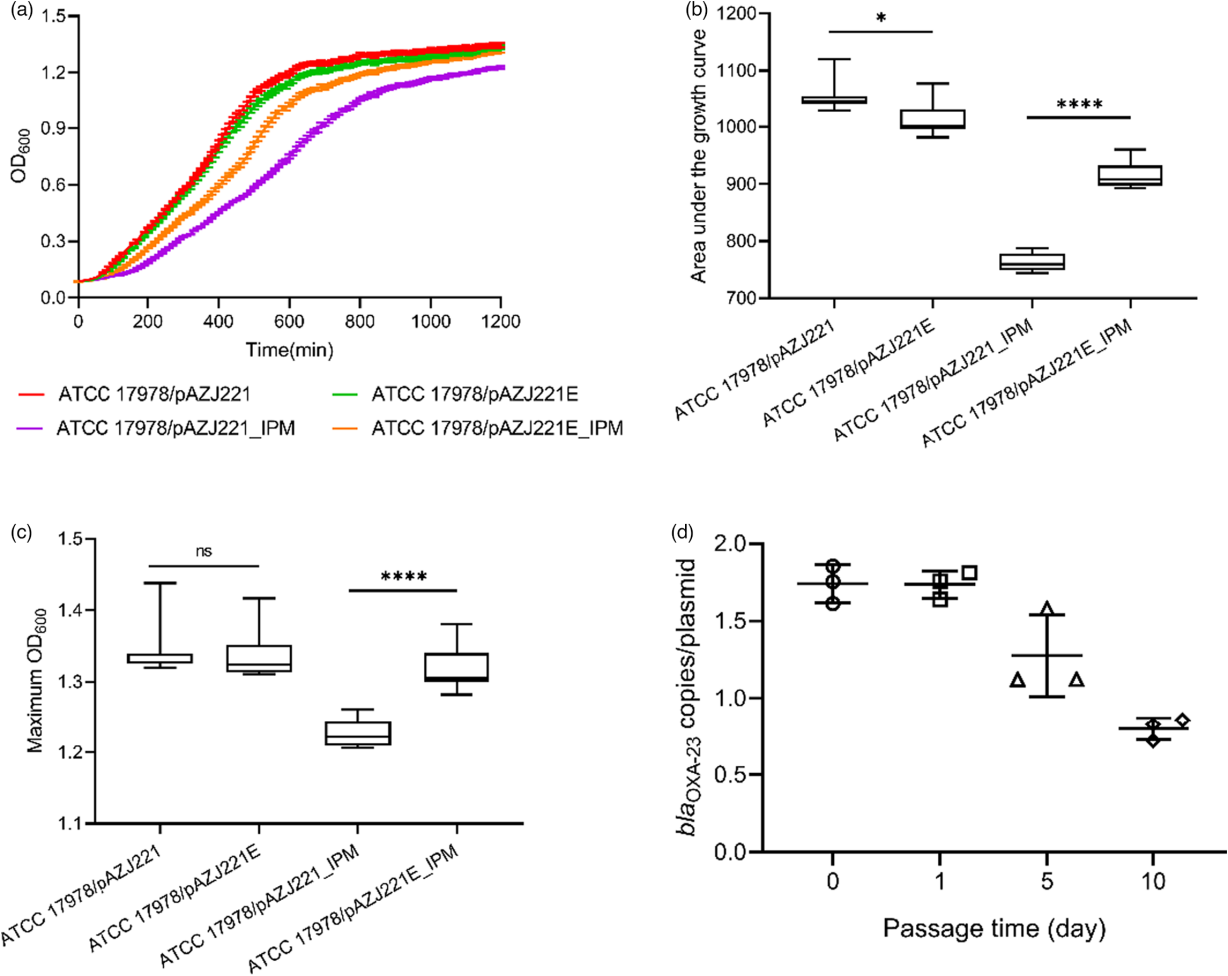
Growth curve (a), the area under the curve (b), and the maximum OD_600_ (c) of ATCC 17978/pAZJ221 and ATCC 17978/pAZJ221E in MH broth and 16 mg/L imipenem MH broth (IPM) are analyzed and compared. Statistical analyses were performed through unpaired *t*‐tests. When the data did not follow normal distribution, Mann–Whitney test was performed. *p < 0.05; ****p < 0.0001; ns, not statistically significant. (d) *bla*_OXA–23_ copy number per plasmid in ATCC 17978/pAZJ221E on four time points during propagation in antibiotic‐free MH broth. The copy number is monitored using qPCR, and three biological replicates are included. Error bars depict the standard deviation of the mean, and the dots represent the data of each replicate

**FIGURE 9:**
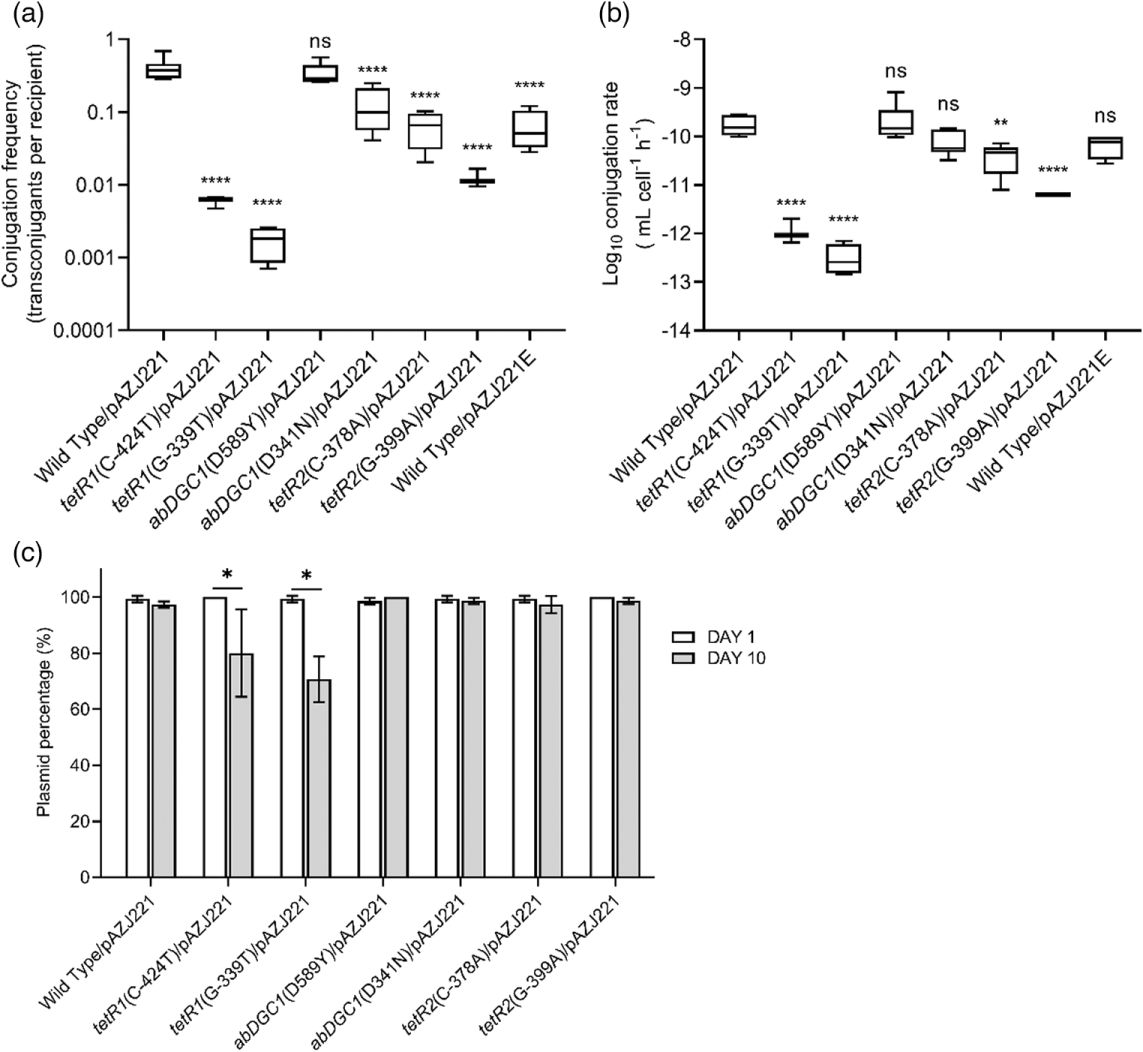
Conjugation frequencies (a) and log_10_conjugation rates (b) of the ancestor strain ATCC 17978/pAZJ221, six mutants with pAZJ221, and ATCC 17978/pAZJ221E. The data of each mutant were compared to that of the ancestor strain ATCC 17978/pAZJ221. Statistical analysis was performed through Dunnett's multiple comparisons test. **, adjusted *p* value < 0.01; ****, adjusted *p* value < 0.0001; ns, not statistically significant. (c) The plasmid‐containing cells percentage of ATCC 17978/pAZJ221 and six mutants with pAZJ221 were assessed on the first day and the tenth day of propagation. Statistical analyses were performed through unpaired *t*‐tests. *p < 0.05; Wild Type, ATCC 17978

Two colonies were selected from two different time points (day 10 and day 60) in lineage 2 and sequenced on the Oxford Nanopore platform. Their assembled pAZJ221 sequences, which we named pAZJ221E, confirmed the existence of two copies of *bla*_OXA−23_. The second copy of *bla*_OXA−23_ had arisen through partial duplication of the original Tn*2009*, resulting in a second copy of the passenger segment and a third copy of IS*Aba1* at the same position as the original insertion (Figure 7). We introduced pAZJ221E into the wild‐type ATCC 17978 by electroporation, verified the presence of two *bla*_OXA−23_ copies by qPCR, and compared the fitness of ATCC 17978/pAZJ221 and ATCC 17978/pAZJ221E in the antibiotic‐free MH broth and 16 mg/L imipenem MH broth. We observed that the evolved plasmid conferred advantages to its host under imipenem pressure, although the burden of pAZJ221E was greater than unevolved pAZJ221 under antibiotic‐free conditions (Figure 8a–c). This suggests that the presence of two *bla*_OXA−23_ copies might have had a positive impact on the fitness of evolving populations at the early stages of the evolution experiment. We also confirmed that the second *bla*_OXA−23_ copy was unstable in an antibiotic‐free environment, as the copy number of *bla*_OXA−23_ gradually decreased to 0.801 ± 0.072 per plasmid after propagation in antibiotic‐free MH broth for 10 days (Figure 8d). Although duplication of *bla*_OXA−23_ increased host fitness under carbapenem pressure, the horizontal transfer ability of pAZJ221E was impaired, as its conjugation frequency was lower than pAZJ221 (Figure 9a).

### 3.9 Changes in plasmid kinetics

Plasmid kinetics are widely assumed to influence the spread of antibiotic resistance genes in bacterial populations and ecosystems (Campos et al., 2020). The average copy number of pAZJ221 in the ancestral host was 1.5 copies per chromosome (1.50 ± 0.17). We determined the copy number of pAZJ221 in six constructed mutants and the copy number of pAZJ221E in wild‐type ATCC 17978. All strains carried around 1.5 copies of pAZJ221 or pAZJ221E per cell (Table S8).

Conjugation is also known to be energetically expensive and entails a reduction in bacterial host fitness, thus reducing the rate of vertical plasmid transmission (San Millan & MacLean, 2017). Statistically significant reduced conjugation rates or conjugation frequencies of pAZJ221 were observed when pAZJ221 was electroporated into the constructed mutants except for *abDGC1*(D589Y) (Figure 9a,b). The result indicated that a decreased conjugation ability was a common evolutionary trend for the evolution of ATCC 17978/pAZJ221.As for the plasmid stability of mutant/pAZJ221combinations, after 10‐day propagation (about 100 generations) only mutants harboring mutations upstream of *tetR1* showed significantly lower plasmid percentages than that on the first day (Figure 9c).

## 4 DISCUSSION

We observed that two mutations upstream of *tetR1* could improve fitness in a new environment where neither pAZJ221 nor imipenem pressure was present, suggesting that these SNPs had pleiotropic fitness effects (Kinsler et al., 2020). Improved fitness in multiple conditions could favor the success of strains and prevent their loss within populations. Strains with the mutations *tetR1*(C‐424T) and *tetR1*(G‐339T) might be well‐suited to respond to future evolutionary challenges and to serve as stable donors or recipients of plasmids such as pAZJ221.

Gene duplication is a major source of evolutionary innovation (Toll‐Riera et al., 2016). Our results emphasized the importance of gene instability in rapidly tuning levels of gene expression, as noted in other studies (Girgis et al., 2021; Tomanek et al., 2020). Fitness advantages resulting from *bla*_OXA−23_ duplication were observed soon after pAZJ221 entered a new host exposed to a carbapenem, and this advantage might facilitate population expansion for the accumulation of chromosomal mutations that stabilize the host‐plasmid pair.

We noted that plasmid fitness and host fitness are not necessarily aligned. Among five mutations that conferred fitness advantages in the presence of pAZJ221 under imipenem pressure, four impaired the transfer ability of pAZJ221 and two decreased plasmid stability (Table 3). A reduced conjugation rate is associated with plasmid fitness cost amelioration, representing a shift toward higher investment in vertical transmission (Dahlberg & Chao, 2003; Harrison & Brockhurst, 2012; Turner et al., 1998). Our results are in line with the work of Hall et al. whose model predicted that amelioration is a more successful strategy than high conjugation rates, and that established bacteria‐plasmid relationships would ultimately trend toward low conjugation rates, because high conjugation rates are only transiently beneficial (Hall et al., 2017).

We propose a potentially general mechanism of *A. baumannii* adaptations to the OXA‐23‐producing plasmid pAZJ221. First, duplication of *bla*_OXA−23_ conferred an immediate advantage in the presence of a carbapenem. Next, chromosomal mutations with diverse effects appeared. Five out of six parallel mutations were verified to confer fitness advantages in the presence of pAZJ221 under imipenem pressure, and all but one of them impaired the plasmid's conjugation ability.

There are some limitations for translating our findings to real‐world situations. First, our study was conducted under laboratory conditions while in the wild, pathogens exist in more complex environments. Additionally, the mechanisms responsible for beneficial effects on the fitness of the mutations require further investigation. Finally, more clinically relevant *A. baumannii* strains should be considered for further evolutionary experiments.

Despite the aforementioned limitations, our study sheds new light on the co‐evolution of an OXA‐23‐producing plasmid that co‐evolves with a new *A. baumannii* host under sustained, sub‐inhibitory carbapenem exposure. To combat the spread and persistence of CRAB, novel therapeutic approaches that target mechanisms contributing to the emergence and dissemination of successful CRAB strains are urgently required. It is also crucial that the ongoing evolution of CRAB and associated carbapenem resistance plasmids are monitored in clinical environments globally.

## CONFLICT OF INTEREST

None to declare.

## DATA AVAILABILITY STSTEMENTS

Sequencing data were deposited to NCBI as Bioproject PRJNA660930 (https://www.ncbi.nlm.nih.gov/bioproject/PRJNA660930). The GenBank accession number of pAZJ221E is CP061542.1 (https://www.ncbi.nlm.nih.gov/nuccore/CP061542). Other raw data for this study are available at Dryad (https://doi.org/10.5061/dryad.05qfttf4k).

## Supporting information


Figure S1
Figure S2.
**Figure S3**.
Figure S4.
Figure S5.
Figure S6.

Appendix S1
