# Supplementary material for: Co‐evolutionary adaptations of Acinetobacter baumannii and a clinical carbapenemase‐encoding plasmid during carbapenem exposure
Source: Evol Appl. 2022 Jul 5;15(7):1045–61. doi: 10.1111/eva.13441 (PMC9309461; doi:10.1111/eva.13441)
Supplement: Supplementary file 1 — Figure S1 Figure S2. Figure S3. Figure S4. Figure S5. Figure S6. [file EVA-15-1045-s001.docx]

**Supplementary Materials**

**Figure legends**

**Fig. S1** The broth cultures of ATCC 17978 in MH broth, ATCC 17978/pAZJ221 and evolving populations from the experimental group in imipenem-containing MH broth. The cultures were under shaking (200 rpm) at 37 °C overnight.

**Fig. S2** Allele frequency dynamics in each lineage during evolution. (A) to (C) represents lineage 1-3, respectively. X-axis represents the 6 time-points detected in the evolution progress and Y-axis represents the frequency (%) of the allele. Different mutations on the same gene or intergenic region are marked with the same color. Genes are presented in the form of locus_tag. The reference sequence ATCC 17978-mff was downloaded from NCBI (CP012004.1).

**Fig. S3** (A) The location of sRNA66, sRNA67, and two mutations at the upstream region of ACX60_11780 (*tetR1*). (B) The predicted secondary structures of wild-type sRNA66 and mutated sRNA66. (C) The predicted secondary structures of wild-type sRNA67 and mutated sRNA67. Mutated positions and their consequences are illustrated. The structures are presented in colors. Green: stems (canonical helices); red: multiloops (junctions); yellow: interior loops; blue: hairpin loops; orange: 5' and 3' unpaired region.

**Fig. S4** (A) The location of sRNA33, and two mutations at the upstream region of ACX60_07155 (*tetR2*). (B) The predicted secondary structures of wild-type sRNA33 and mutated sRNA33. Mutated positions and their consequences are illustrated. The structures are presented in colors. Green: stems (canonical helices); red: multiloops (junctions); yellow: interior loops; blue: hairpin loops; orange: 5' and 3' unpaired region.

**Fig. S5** COG categories of genes targeted by sRNA33 (A), sRNA66 (B) and sRNA67 (C).The letters represent different COG categories. C, energy production and conversion; D, cell cycle control, cell division, chromosome partitioning; E, amino acid transport and metabolism; F, nucleotide transport and metabolism; G, carbohydrate transport and metabolism; H, coenzyme transport and metabolism; I, lipid transport and metabolism; J, translation, ribosomal structure and biogenesis. K, transcription; L, replication, recombination and repair; M, cell wall/membrane/envelope biogenesis; O, posttranslational modification, protein turnover, chaperones; P, inorganic ion transport and metabolism; Q, secondary metabolites biosynthesis, transport and catabolism; S, function unknown; V, defense mechanisms.

**Fig. S6** The effect of D94N mutation on the protein structure of ACX60_10935. The signal peptide (the first 26 residues) was removed from the amino acid sequence. AlphaFold 2.0 was used to predict the protein structure of ACX60_10935 wild type protein and D94N mutation protein. Green: wild type ACX60_10935; Cyan: ACX60_10935 D94N.

**Fig. S1**


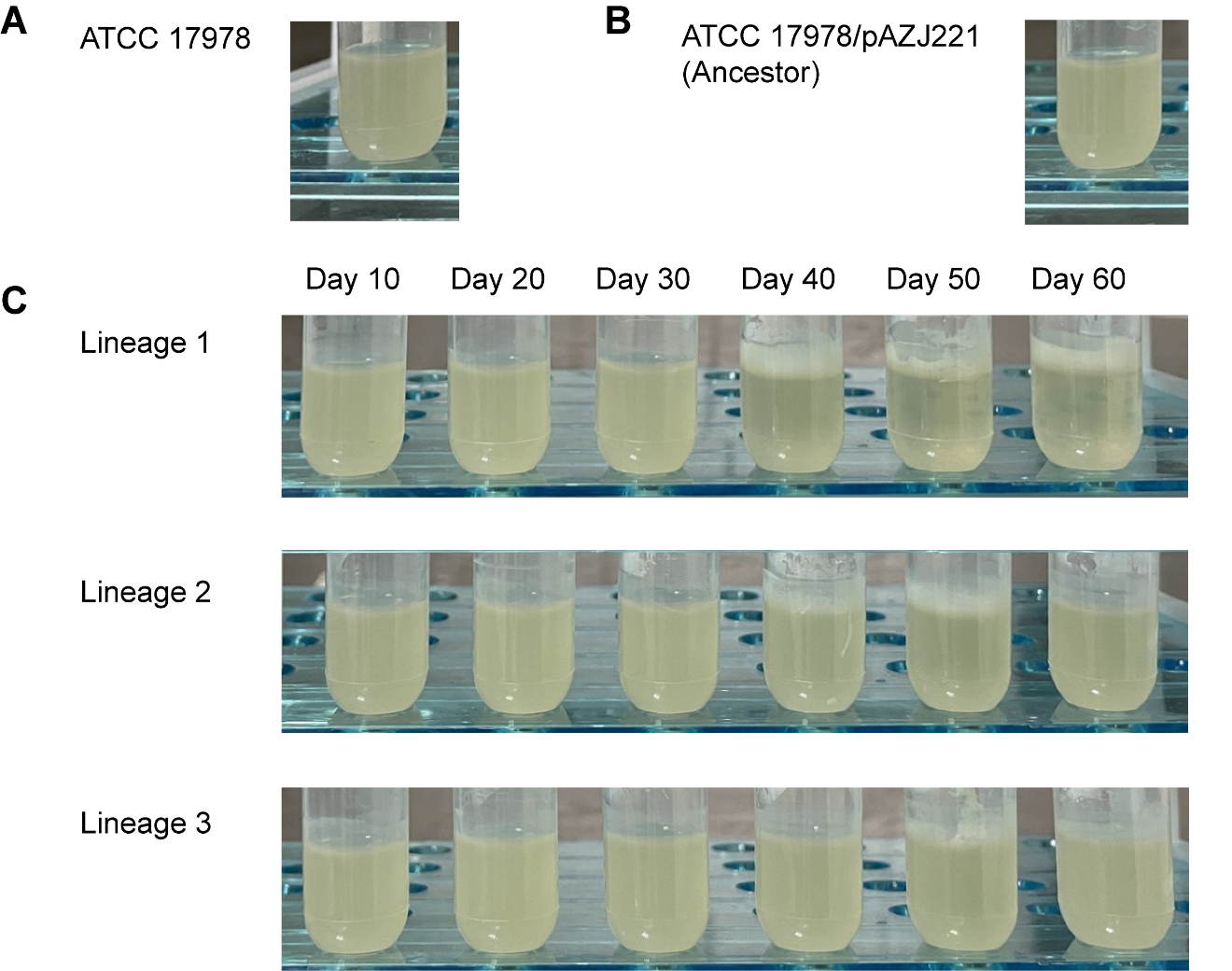


**Fig. S2**


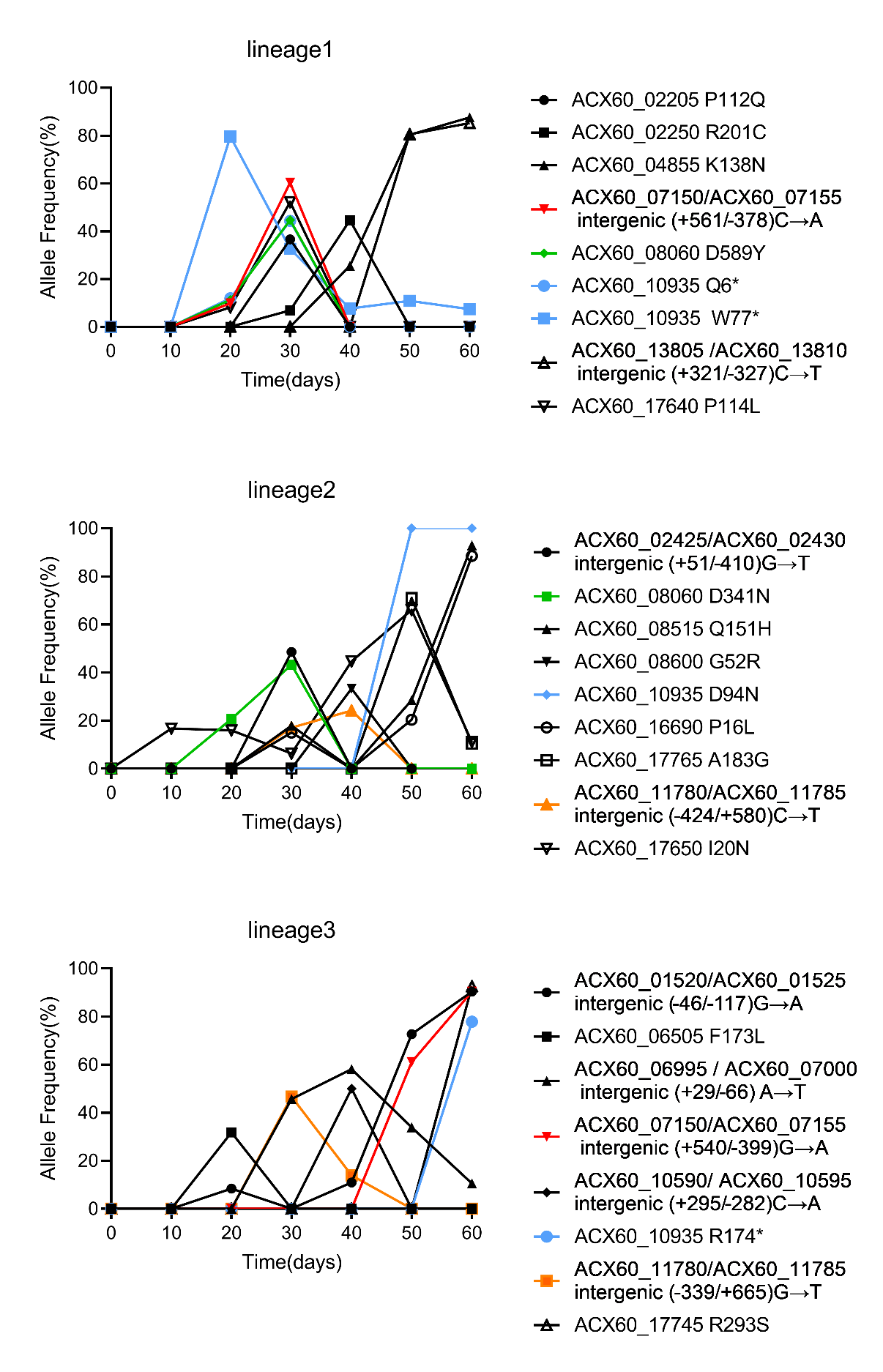


**Fig. S3**


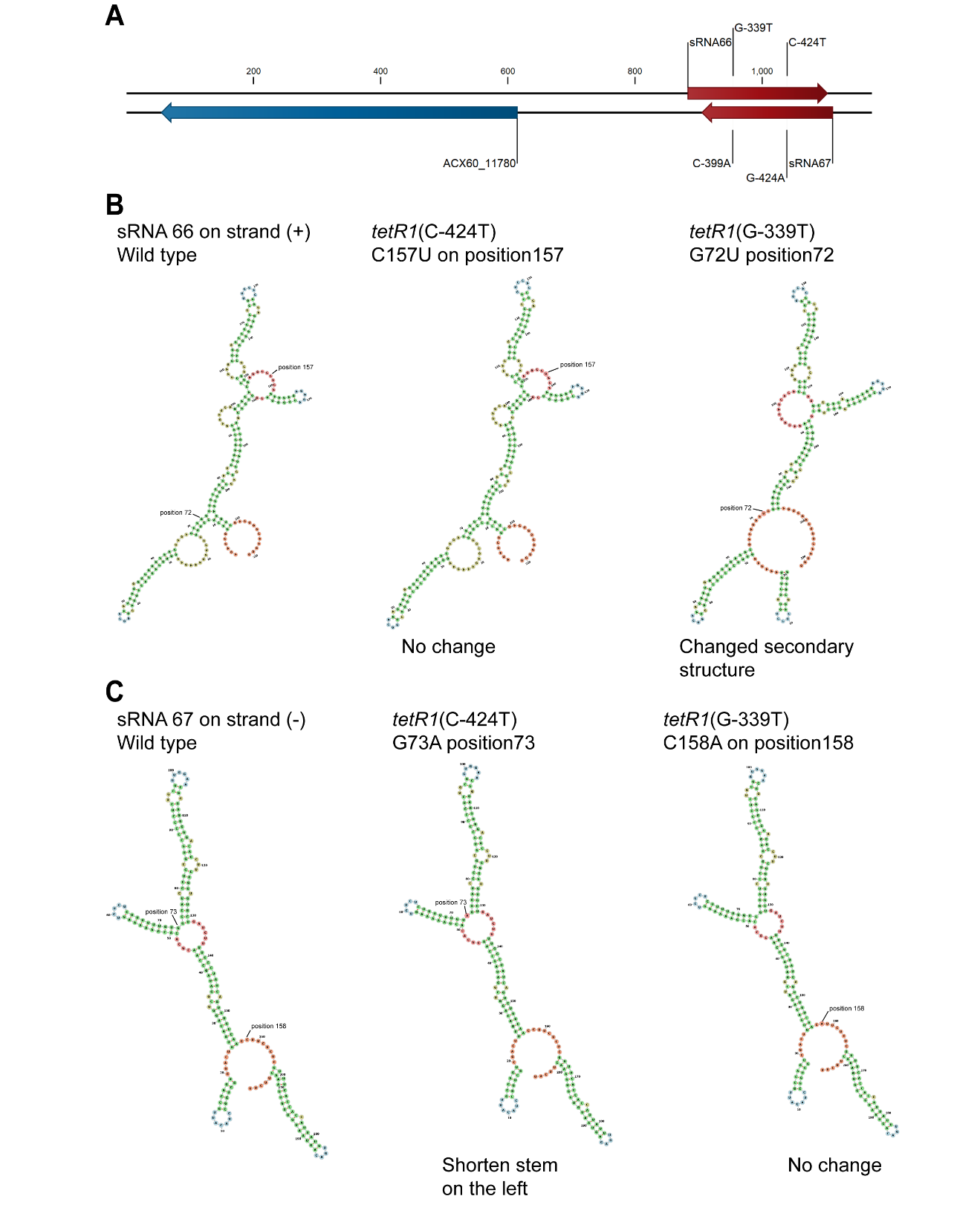


**Fig. S4**


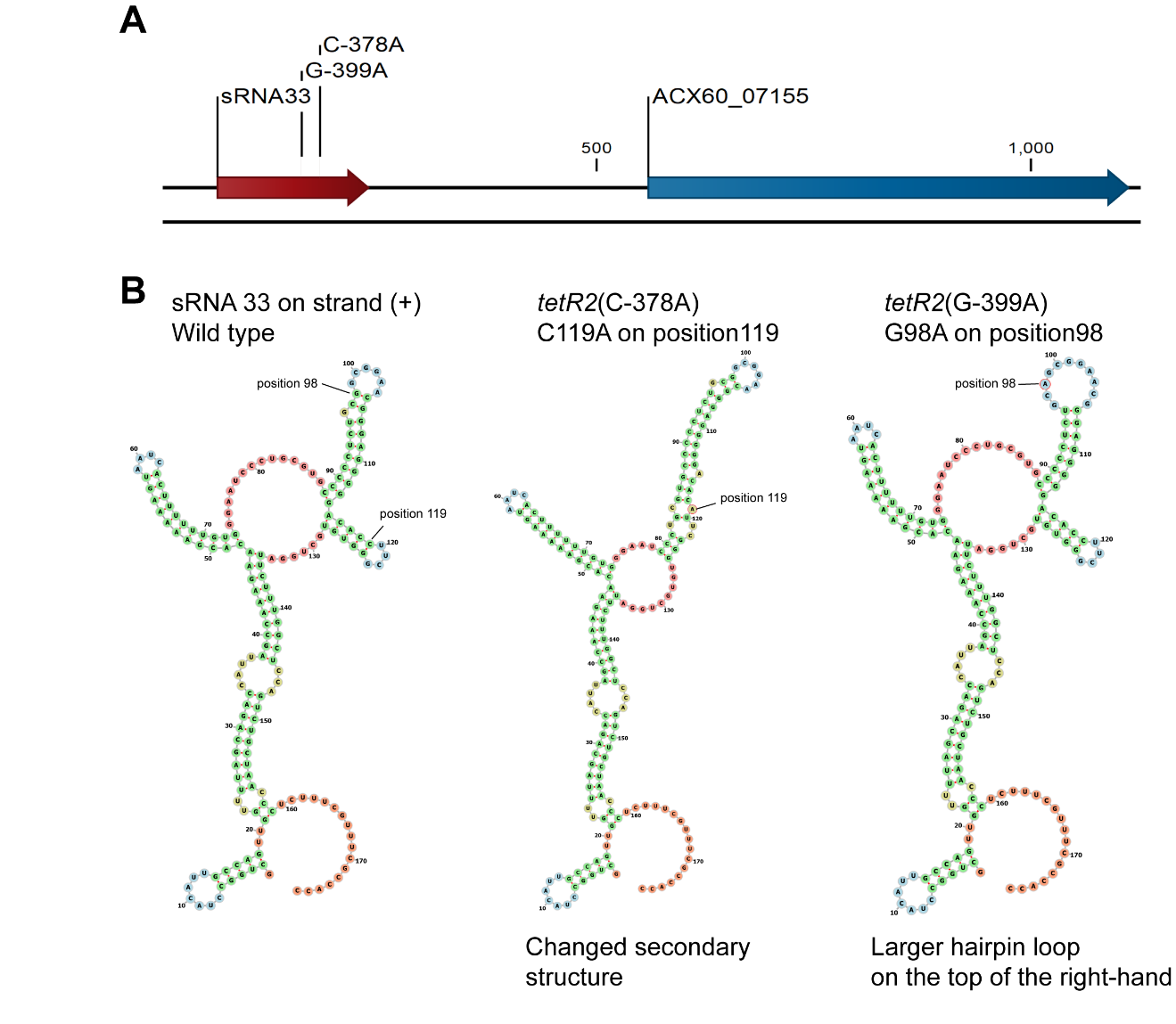


**Fig. S5**


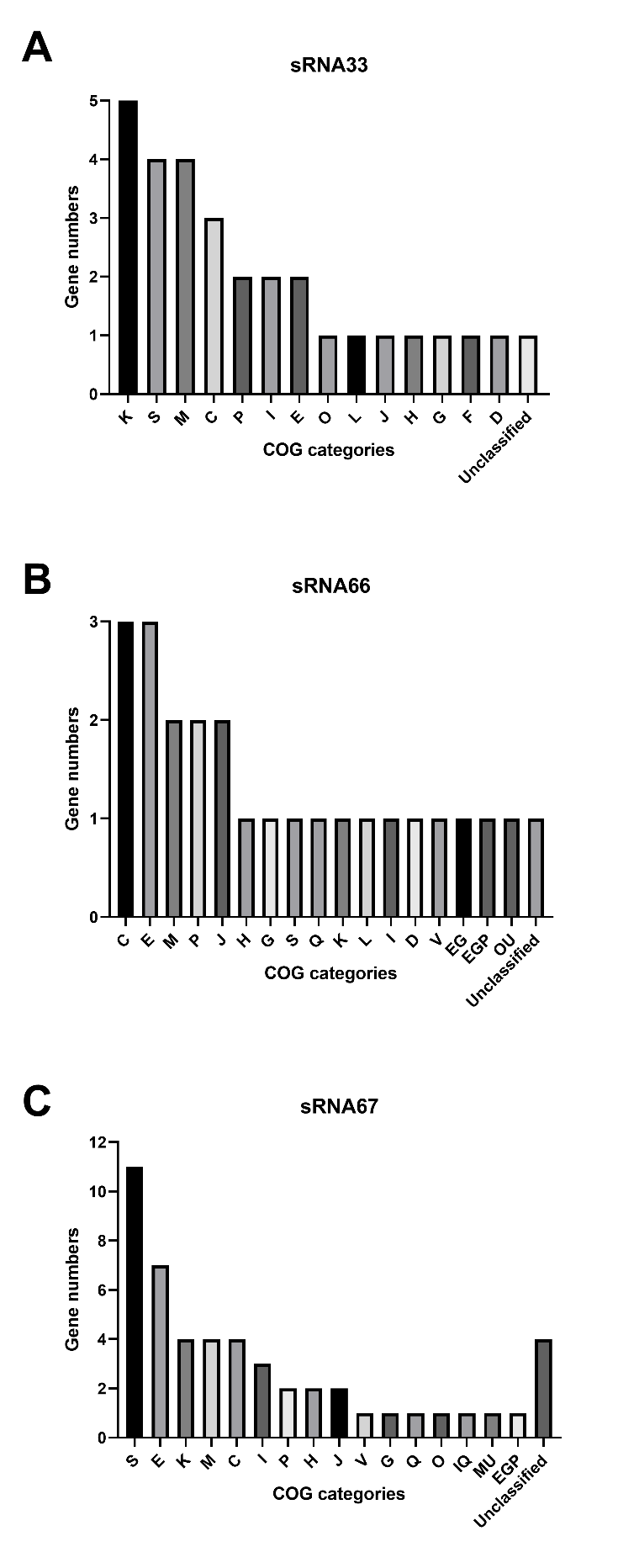


**Fig. S6**

**
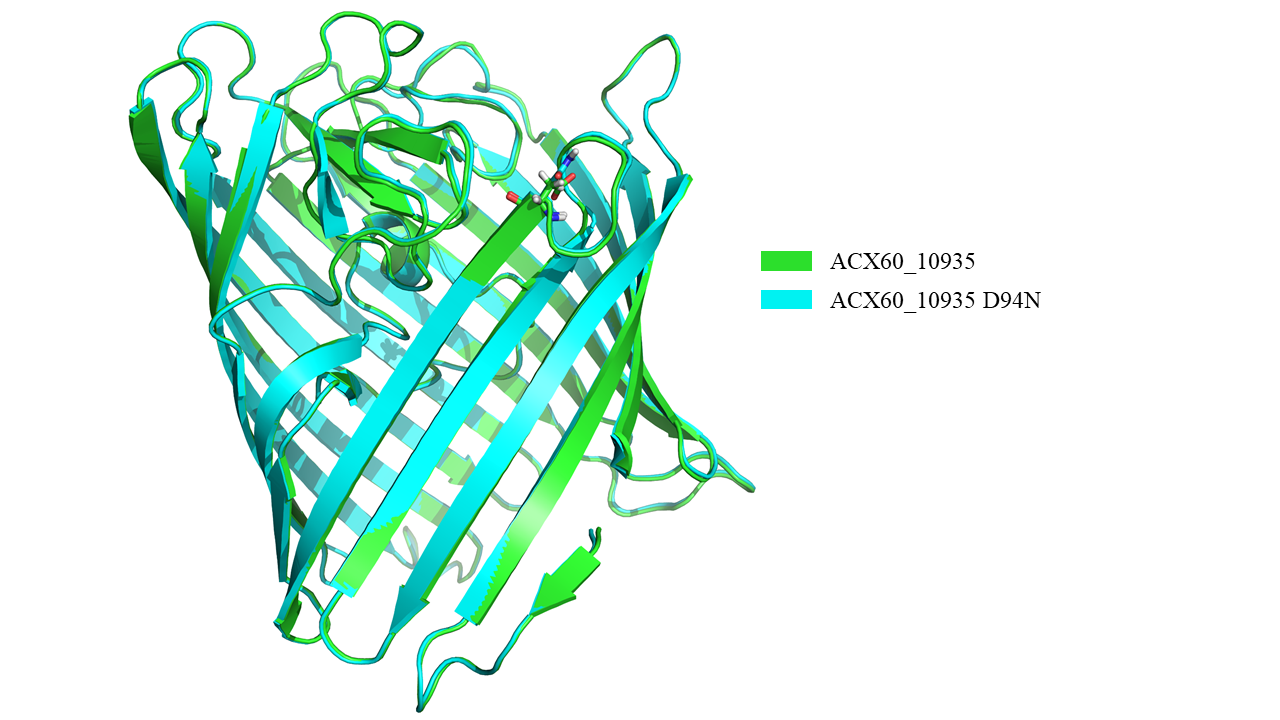
**
