# Supplementary material for: Co‐evolutionary adaptations of Acinetobacter baumannii and a clinical carbapenemase‐encoding plasmid during carbapenem exposure
Source: Evol Appl. 2022 Jul 5;15(7):1045–61. doi: 10.1111/eva.13441 (PMC9309461; doi:10.1111/eva.13441)
Supplement: Supplementary file 2 — Appendix S1 [file EVA-15-1045-s002.zip › EVA_13441_Suppl_Table_0513.docx]

| Lineage | Time-point of storage | Average coverage on chromosome | Average coverage on pAB1 | Average coverage on pAB2 | Average coverage on pAB3 | Average coverage on pAZJ221 |
| --- | --- | --- | --- | --- | --- | --- |
| 1 | Day10 | 264x | 2865x | 5339x | 347x | 382x |
| 1 | Day20 | 323x | 2773x | 4952x | 403x | 509x |
| 1 | Day30 | 189x | 422x | 640x | 138x | 235x |
| 1 | Day40 | 205x | 571x | 888x | 269x | 246x |
| 1 | Day50 | 238x | 660x | 1002x | 298x | 252x |
| 1 | Day60 | 270x | 521x | 791x | 324x | 245x |
| 2 | Day10 | 269x | 2220x | 4299x | 308x | 381x |
| 2 | Day20 | 336x | 2649x | 4784x | 469x | 444x |
| 2 | Day30 | 293x | 891x | 1437x | 242x | 312x |
| 2 | Day40 | 226x | 486x | 673x | 455x | 334x |
| 2 | Day50 | 220x | 721x | 940x | 522x | 378x |
| 2 | Day60 | 253x | 408x | 666x | 312x | 235x |
| 3 | Day10 | 305x | 3243x | 6044x | 408x | 418x |
| 3 | Day20 | 295x | 3047x | 5881x | 424x | 427x |
| 3 | Day30 | 274x | 610x | 992x | 186x | 284x |
| 3 | Day40 | 287x | 789x | 1259x | 133x | 301x |
| 3 | Day50 | 253x | 726x | 1095x | 211x | 252x |
| 3 | Day60 | 254x | 506x | 783x | 270x | 223x |

Table S1 Reads coverage of evolving populations from the experimental group

| Name | Primer Sequence(5’-3’) | Gene/Region | Use |
| --- | --- | --- | --- |
| D589Y F | CAACAACTCAATCAACCC | ACX60_08060 | mutation verification |
| D589Y R | GCAATAAGTGAAGTAGGAGA |  |  |
| D341N F | CTTTTACCACCCCATATTC | ACX60_08060 | mutation verification |
| D341N R | TAGAGAAACAGGAAGCGG |  |  |
| GT F | TCTCGGGTTTATTGTTGCT | ACX60_07150/ ACX60_07155 | mutation verification |
| GT R | AGAGCGGAAGAAAGAGGA |  |  |
| 11780-F | CAGGCATGTAAAAGCTGCAC | ACX60_11780/ACX60_11785 | mutation verification |
| 11780-R | TCCTTTCTTTCTGGTAGAGTGA |  |  |
| 10935-F | AGAAGTCCCTGACATCCCCC | ACX60_10935 | mutation verification |
| 10935-R | GTATGAGCACCGCGAACAAC |  |  |
| rep221-F | ATCACACTCGCACATACA | *repA* | plasmid verification |
| rep221-R | GCCGTCTTTTTTCCAACA |  |  |
| DY-C-F | cggccgccctgcagcggatccCCCCAATTCAACATACAAAC | ACX60_08060 | mutant construction |
| DY-C-R | agcccgtcgcatgcatctagaATACGGGAGGCAAAAAAG |  |  |
| DN-C-F | cggccgccctgcagcggatccGAACACTACCCAAAAGCA | ACX60_08060 | mutant construction |
| DN-C-R | agcccgtcgcatgcatctagaTCTTCTTGTTCTGGTGTTTC |  |  |
| GT-C-F | cggccgccctgcagcggatccGAAACTTGGACAAACCGT | ACX60_07150/ ACX60_07155 | mutant construction |
| GT-C-R | agcccgtcgcatgcatctagaAGCGCTCATCTTTCCAAA |  |  |
| 11780-C-F | cggccgccctgcagcggatccCAGGCATGTAAAAGCTGCAC | ACX60_11780/ACX60_11785 | mutant construction |
| 11780-C-R | agcccgtcgcatgcatctagaTCCTTTCTTTCTGGTAGAGTGA |  |  |

Table S2 Primers used for mutation verification and construction.

Table S3 Primers used for qPCR.

| Primers | | PCR description | | |
| --- | --- | --- | --- | --- |
| Name | Sequence (5’-3’) | Target | Size (bp) | Efficiency and R^2^*^a^* |
| rpoD_mff_F | ACATGGCCGTAATAGCCCTGAA | *rpoD* | 180 | 97.5209% |
| rpoD_mff_R | ATACGGCCACGACGTACTGC |  |  | 0.9989 |
| repA_mff_F | GGCCGCAATGACCATCTTTTT | *repA* | 180 | 96.6736% |
| repA_mff_R | TATCGACTTGGCCGTTGCTTT |  |  | 0.999 |
| OXA23_mff_F | AATGGAAGGGCGAGAAAAGGTC | *bla*_OXA-23_ | 180 | 97.7759% |
| OXA23_mff_R | CAACCTGCTGTCCAATTTCAGC |  |  | 0.9993 |

*^a^* R^2^, correlation coefficient of the standard curve for the efficiency.

Table S4 Conjugation frequency of *Acinetobacter* plasmids

| Plasmid | Size  (bp) | Donor | Receptor | Conjugation frequency | Reference |
| --- | --- | --- | --- | --- | --- |
| pAZJ221 | 77,530 | *A. baumannii*  ATCC 17978 | *A. baumannii* ATCC 17978 Rif^R^ | 4.08×10^-1^  (per recipient) | this study |
| pALWED1.1 | 287,631 | *A. lwoffii* ED23-35 | *A. baylyi* BD413rif | 8×10^-3^ (per recipient) | (1) |
| pFM-M19 | 55,044 | *A. johnsonii* M19 | *E. coli* 25DN | 1.6×10^–4^ (per donor) | (2) |
| pDETAB2 | 100,072 | *A. baumannii* DETAB-P2 | *A. baumannii* ATCC 17978 Rif^R^ | 2.35×10^-6^ (per donor) | (3) |

(1) Mindlin S, et al. (2021) Ubiquitous Conjugative Mega-Plasmids of *Acinetobacter* Species and Their Role in Horizontal Transfer of Multi-Drug Resistance. Front Microbiol 12:728644.

(2) Zong G, et al. (2020) The carbapenem resistance gene *bla*_OXA-23_ is disseminated by a conjugative plasmid containing the novel transposon Tn*6681* in *Acinetobacter johnsonii* M19. Antimicrobial resistance and infection control 9(1):182.

(3) Liu H, et al. (2021) Transferable *Acinetobacter baumannii* plasmid pDETAB2 encodes OXA-58 and NDM-1 and represents a new class of antibiotic resistance plasmids. J Antimicrob Chemother 76(5):1130-1134.

Table S7 Carbapenem MICs (mg/L) of ATCC 17978, the ancestral strain and constructed mutants with pAZJ221

|  | MIC (mg/L) | |
| --- | --- | --- |
| Strain | Imipenem | Meropenem |
| ATCC 17978 | 0.5 | 0.5 |
| ATCC 17978/pAZJ221 | 32 | 64 |
| *tetR1*(C-424T)/pAZJ221 | 32 | 64 |
| *tetR1*(G-339T) /pAZJ221 | 32 | 64 |
| *abDGC1*(D589Y) /pAZJ221 | 64 | 64 |
| *abDGC1*(D341N) /pAZJ221 | 64 | 64 |
| *tetR2*(C-378A) /pAZJ221 | 32 | 64 |
| *tetR2*(G-399A) /pAZJ221 | 32 | 64 |

Table S8 Plasmid copy number of pAZJ221 and pAZJ221E in strains

| Strain | Plasmid copy number  ( copies per cell) | Adjusted *P* value^a^ |
| --- | --- | --- |
| ATCC 17978/pAZJ221 | 1.50 ± 0.17 | NA^b^ |
| *tetR1*(C-424T)/pAZJ221 | 1.38±0.05 | 0.2674 |
| *tetR1*(G-339T)/pAZJ221 | 1.22±0.02 | ≤0.05 |
| *abDGC1*(D589Y)/pAZJ221 | 1.38±0.08 | 0.3217 |
| *abDGC1*(D341N)/pAZJ221 | 1.35±0.05 | 0.1201 |
| *tetR2*(C-378A)/pAZJ221 | 1.29±0.01 | ≤0.05 |
| *tetR2*(G-399A)/pAZJ221 | 1.17±0.04 | ≤0.001 |
| ATCC 17978/pAZJ221E | 1.86±0.06 | ≤0.001 |

^a^Adjusted *P* value was calculated using Dunnett's multiple comparisons test, the plasmid copy number of each mutant was compared with that of ATCC 17978/pAZJ221.

^b^NA, not applicable.
